# A Multi-Modal Graph Neural Network Framework for Parkinson’s Disease Therapeutic Discovery

**DOI:** 10.3390/ijms26094453

**Published:** 2025-05-07

**Authors:** Ömer Akgüller, Mehmet Ali Balcı, Gabriela Cioca

**Affiliations:** 1 Faculty of Science, Department of Mathematics, Mugla Sitki Kocman University, Mugla 48000, Turkey; oakguller@mu.edu.tr; 2 Faculty of Medicine, Preclinical Department, Lucian Blaga University of Sibiu, 550024 Sibiu, Romania; gabriela.cioca@ulbsibiu.ro

**Keywords:** Parkinson’s disease, drug repurposing, graph neural network, protein–protein interaction, polypharmacology

## Abstract

Parkinson’s disease (PD) is a complex neurodegenerative disorder lacking effective disease-modifying treatments. In this study, we integrated large-scale protein–protein interaction networks with a multi-modal graph neural network (GNN) to identify and prioritize multi-target drug repurposing candidates for PD. Network analysis and advanced clustering methods delineated functional modules, and a novel Functional Centrality Index was employed to pinpoint key nodes within the PD interactome. The GNN model, incorporating molecular descriptors, network topology, and uncertainty quantification, predicted candidate drugs that simultaneously target critical proteins implicated in lysosomal dysfunction, mitochondrial impairment, synaptic disruption, and neuroinflammation. Among the top hits were compounds such as dithiazanine, ceftolozane, DL-α-tocopherol, bromisoval, imidurea, medronic acid, and modufolin. These findings provide mechanistic insights into PD pathology and demonstrate that a polypharmacology approach can reveal repurposing opportunities for existing drugs. Our results highlight the potential of network-based deep learning frameworks to accelerate the discovery of multi-target therapies for PD and other multifactorial neurodegenerative diseases.

## 1. Introduction

Parkinson’s disease is a complex neurodegenerative disorder characterized by multifactorial etiology and the dysregulation of numerous cellular pathways [1,2,3]. The absence of disease-modifying therapies for PD, despite intensive research, underscores the need for new approaches that can disentangle this complexity and reveal novel therapeutic targets [4]. In this context, a systems biology perspective—integrating large-scale molecular data—and network science—analyzing the web of protein interactions—offers a powerful framework to interrogate disease mechanisms. Protein–protein interaction (PPI) networks, which map the interactome of the cell, have emerged as invaluable tools for modeling the molecular crosstalk underlying PD pathogenesis [5,6,7]. By examining the topological features of these networks, such as node centrality and clustering, researchers can identify key molecular hubs and modules that drive disease processes and are attractive targets for drug discovery.

Recent advances in network medicine illustrate the promise of this approach. Network-based analyses systematically capture the interconnections between genes, proteins, and pathways in disease, enabling the identification of “disease modules”—groups of functionally related, interacting molecules that collectively contribute to pathology [8,9,10]. PD, like many complex diseases, is thought to result from perturbations in such network modules rather than a single causative gene. Indeed, therapeutic strategies that target dysregulated pathways or processes, rather than individual proteins, may prove more effective for complex disorders [11,12,13]. This shift from one gene–one drug paradigms toward multi-target, network-level interventions is supported by the intrinsic redundancy and robustness of biological networks [14,15]. In PD, for example, distinct genetic or environmental triggers often converge on common pathways (mitochondrial dysfunction, proteostasis, neuroinflammation, etc.), forming interlinked clusters of proteins that collectively drive neurodegeneration [16,17]. Understanding the PPI network topology of PD can thus provide mechanistic insights into these pathogenic clusters and reveal pivotal nodes within them.

One key topological concept is node centrality, which quantifies a protein’s importance or influence in the network. Classic centrality measures–degree, betweenness, closeness, eigenvector centrality, among others–have long been used in systems biology to pinpoint influential nodes in molecular networks [18,19,20]. High-degree hub proteins, for instance, often correspond to essential genes or master regulators, as observed in yeast and other model organisms. In the human interactome, hubs are frequently critical for cell viability and are enriched for disease-related genes. PD-specific PPI networks exemplify this principle: analyses of familial PD genes show that proteins like Leucine-Rich Repeat Kinase 2 (LRRK2) occupy hub positions with exceptionally high connectivity and betweenness centrality [21]. LRRK2 and other PD-associated hubs participate in multiple PD-relevant processes (e.g., autophagy, vesicle transport), linking various disease modules. Notably, bottleneck nodes with high betweenness (even if not highest in degree) can serve as critical bridges between modules, sometimes even surpassing hubs in correlating with essential functions. By mapping out these central nodes, researchers can prioritize candidates that orchestrate network-wide dysfunction in PD.

Another informative topological feature is the presence of clustered communities or functional modules within the PPI network. Clustering algorithms and clique detection can uncover tightly knit groups of proteins–often reflecting biochemical pathways or complexes–that are collectively perturbed in disease. In PD, known gene products tend to coalesce into a few major network communities related to mitochondrial quality control, synaptic transmission, and protein aggregation pathways [22,23,24]. For example, Rakshit et al. [25] constructed PD-specific PPI networks from gene expression data and identified several cliques (fully connected sub-networks) enriched in PD-related proteins. These cliques constituted core functional modules of the PD network, each representing a critical biological process, and contained most of the topologically significant nodes (hubs and bottlenecks). Strikingly, by focusing on such network modules, the study uncovered 37 previously unreported PD-associated proteins that had not been classical PD genes. Many of these new candidates were centrally positioned in the interactome and showed altered co-expression patterns, suggesting they are part of the PD mechanism. The authors proposed these network-derived proteins as potential PD biomarkers and therapeutic targets, highlighting how PPI network topology can lead to novel hypotheses for intervention.

### Literature Review

A growing body of literature supports the notion that PPI network topology can yield mechanistic insights and drive target discovery in neurodegenerative diseases. Network-based studies in PD have demonstrated multiple applications of PPI analysis. Tomkins and Manzoni [4] reviewed how PPI networks have been used to infer PD-related pathways, prioritize novel disease genes, and compare PD’s network footprint to other neurodegenerative disorders. They emphasized that PPI networks “accelerate our understanding of the molecular crosstalk and biological processes underlying PD pathogenesis” by modeling the disease as a perturbed network rather than isolated factors. PPI analyses serve as a bridge between genetics and functional biology, integrating diverse omics data to build a coherent picture of PD’s molecular landscape. For instance, integrating gene expression profiles with PPIs has led to the identification of dysregulated network modules in post-mortem PD brains [25]. In one such study, differential expression data from PD substantia nigra were overlaid on the human interactome to construct a PD-specific network, from which hub and bottleneck proteins were extracted. This network-based approach revealed novel candidates (e.g., Arrestin Beta 2 (ARRB2), Transferrin Receptor (TFRC)) linked to dopamine signaling that traditional analysis had overlooked. The topologically prominent nodes in the PD network were not only statistically significant but also mapped to known pathogenic processes (such as neurotransmitter regulation and mitochondrial function), lending biological plausibility to their role in PD. Such findings underscore how network topology–through measures like connectivity and clustering–can expose hidden relationships between genes and pathways, offering mechanistic hypotheses for experimental validation.

Crucially, network approaches have moved beyond mere correlation to predictive, mechanistic modeling in PD. Keane et al. [1] provided a compelling example by using PPI networks to identify combination targets for therapeutic intervention. By constructing an interaction network centered on two hallmark PD stressors (mitochondrial dysfunction and autophagy inhibition) in a cellular PD model, they pinpointed four proteins with high network influence in mediating neuronal death. Remarkably, simultaneous modulation of these network targets (through combined knockdowns or overexpression) protected neurons from toxin-induced death, whereas targeting any single protein alone was insufficient. This result validated the concept that PD pathology arises from convergent network effects and that a multi-target strategy informed by network topology can be effective in rescuing disease phenotypes. In essence, the study demonstrated a mechanistic link between network hubs and PD’s cellular pathology, providing proof-of-concept that network analysis can directly inform therapeutic design.

Parallel developments in computational biology have sought to leverage these network insights for drug discovery and repurposing. Traditional in silico drug repurposing methods often use networks in a heuristic way (e.g., network diffusion scores or “guilt-by-association” where a drug’s known targets are connected to disease genes). However, these methods may not fully capture the complexity of network topology [26]. Recent approaches embrace graph-based machine learning, particularly Graph Neural Networks (GNNs), to learn from biomedical networks in a more nuanced manner [27,28,29]. GNNs are a class of deep learning models designed to operate on graph-structured data, propagating information along edges to create low-dimensional embeddings that encode each node’s local neighborhood and global position in the network [30,31]. By iterative message passing, GNNs can incorporate higher-order connectivity patterns and multi-node interactions that elude simpler network metrics. Importantly, applying GNNs to biological networks enables the integration of multimodal data within a unified framework. As Zhou et al. [32] note, “adopting GNN into the biomedical network facilitates the integration of multimodal and complex relationships”, and indeed GNN models have shown great promise in predicting diverse biological interactions–from PPIs and gene–disease links to drug–drug and drug–target interactions. This makes them ideally suited for drug repurposing tasks, where one must analyze a tangle of heterogeneous data (chemical structures, protein networks, disease phenotypes, etc.) [33,34,35].

Several pioneering studies have applied GNNs to drug repurposing with notable success. For example, in [36] researchers have constructed large knowledge graphs (networks with multiple entity types and relations) such as the Drug Repurposing Knowledge Graph (DRKG) that encompass drugs, diseases, genes/proteins, and other biomedical entities. Training graph convolutional networks on these knowledge graphs has led to the discovery of novel drug–disease associations. In one case, a GNN model integrating data from DrugBank, Search Tool for the Retrieval of Interacting Genes/Proteins (STRING), and other databases was able to learn a representation of the polypharmacological network (15 million edges spanning drugs, targets, pathways, and phenotypes) and use it to prioritize candidate treatments for COVID-19. These GNN-driven approaches outperform earlier network-based methods by learning the optimal features and connections from data, rather than relying on manual feature engineering. In a 2024 study, Li et al. [37] introduced a heterogeneous Drug–Target–Disease GNN (DTD-GNN) that combines graph convolution and attention mechanisms; this model achieved higher accuracy (Area Under the Receiver Operating Characteristic Curve (AUC), the harmonic mean of precision and recall (F1-score)) in predicting drug–disease associations compared to conventional techniques, illustrating the power of deep learning on multi-relational graphs. Likewise, Yella et al. [38] developed GraMDTA, a multimodal GNN for drug–target affinity prediction, which learns from both molecular graphs (drug chemical structure) and knowledge graphs (biomedical relationships) to improve prediction robustness. These examples highlight a clear trend: deep geometric learning is revolutionizing computational drug repurposing by merging chemical information with systems-level network data in a single predictive model.

Despite these advances, challenges remain in translating predictions into clinical insight. One crucial aspect is uncertainty quantification (UQ) in model predictions. In high-stakes domains like drug discovery for PD, it is not enough for a model to output a candidate drug–target interaction; researchers and clinicians also need an estimate of confidence in that prediction [39,40]. Standard GNN models, however, often act as “black boxes” that do not report uncertainty, which can undermine trust in their recommendations. Recent work has begun to address this gap. For instance, Jiang et al. [41] introduced an ensemble-based GNN architecture search to quantify both aleatoric and epistemic uncertainty in molecular property predictions, emphasizing that accurate UQ is crucial for decision-making in drug discovery. By obtaining uncertainty measures, one can flag predictions that require further validation or prioritize highly confident drug–gene hits for experimental follow-up. Incorporating UQ thus adds a layer of rigor to computational repurposing pipelines, aligning them with the risk-management practices of pharmaceutical development.

The current study advances the established body of research at the confluence of network biology and graph-based machine learning. We introduce an innovative multi-modal graph neural network architecture that synthesizes heterogeneous biological data modalities—spanning drug physicochemical properties, PD-specific PPI network topology, and cluster-derived functional gene annotations—to predict therapeutically relevant drug–gene interactions in PD. Within this framework, pharmacological agents are characterized by a dual representation encompassing both molecular descriptor profiles and their empirical or inferred interactome dynamics, while genes/proteins are annotated through their topological prominence within the PPI network (via a novel centrality metric) and their affiliation with functional modules (e.g., PD-associated pathways or gene ontology categories). The integration of these modalities enables the GNN to construct a unified embedding space that harmonizes chemical and proteomic interaction landscapes, thereby facilitating sophisticated polypharmacology analysis: the model discerns latent compound–target associations, even in cases where such relationships evade conventional detection. To further enhance interpretability, we embed a probabilistic uncertainty estimation module within the GNN, permitting rigorous quantification of predictive confidence. This holistic system embodies a methodological advancement by merging network science—leveraging systems-level insights into PD pathophysiology—with geometric deep learning techniques, which adeptly model nonlinear dependencies across multifaceted data. We postulate that strategic emphasis on topological features of the PPI network—including hub proteins, bottleneck nodes, and module-specific key network nodes—will orient the model toward biologically salient targets, thereby augmenting the efficacy of PD drug candidate identification. By bridging mechanistic principles of network architecture with cutting-edge graph analytics and principled uncertainty quantification, this work aspires to redefine computational drug repurposing paradigms and deliver a robust, systems biology-driven platform for accelerating PD therapeutic discovery.

## 2. Results and Discussions

### 2.1. Parkinson’s Disease Network

The genome-wide association study (GWAS) dataset for Parkinson’s disease, sourced from the latest database version (30.01.2025), initially encompassed 859 genetic associations. We set the genome-wide significance threshold at $p<5\times 10^{-8}$ because this is the standard in large-scale association studies to account for the roughly one million independent common variants tested across the human genome. Such a stringent cutoff controls the family-wise error rate and minimizes false positives, ensuring that only the most robust locus associations are carried forward into downstream network and enrichment analyses. This filtration step reduced the dataset to 512 high-confidence associations. Further refinement involved eliminating redundant single nucleotide polymorphism (SNP) entries and associations lacking mapped gene annotations, culminating in a final cohort of 140 unique SNPs. These SNPs were subsequently interrogated for functional interactions using the STRING database, which aggregates PPI data to infer molecular relationships. Adhering to standardized protocols for network construction in Cytoscape (v3.4.0), an open-source platform for visualizing and analyzing molecular interaction networks [42], we generated a comprehensive interaction network comprising 140 nodes (each representing a PD-associated SNP) and 2324 edges (reflecting experimentally validated or computationally predicted PPIs). This network served as the foundation for downstream systems-level analysis of PD pathobiology.

**Figure 1:**
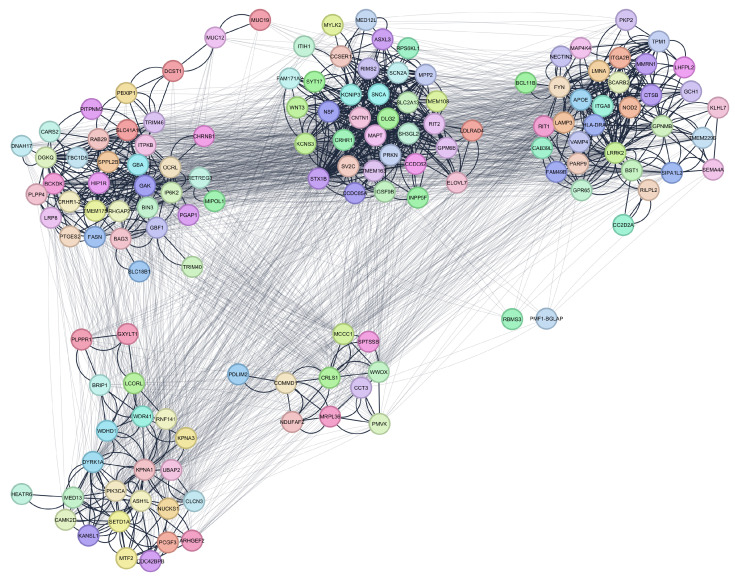
A Clustered network for Parkinson’s Disease related genes. The light edges represent intercluster connections, while the bold edges represent intracluster connections.

To elucidate the biological architecture underpinning PD, the integrated genomic and proteomic network was subjected to advanced clustering analysis. The Leading Eigenvector Algorithm, implemented via the clusterMaker2 plugin in Cytoscape—an add-on that provides a suite of network clustering and community-detection methods [43]—was employed to partition the network into five modules based on gene coexpression patterns. This method leverages spectral graph theory to identify densely interconnected subnetworks, each posited to encapsulate distinct functional or regulatory pathways. The resulting modular structure, visualized in Figure 1, revealed two notable exceptions: the genes Polyamine-Modulating Factor 1–Bone Gamma-Carboxyglutamate Protein (PMF1-BGLAP) and RNA Binding Motif Single-Stranded Interacting Protein 3 (RBMS3) exhibited lower modularity scores, indicating weaker affiliation with their respective clusters. However, given their biological relevance—PMF1-BGLAP’s role in osteoblast differentiation and potential cross-talk with neurodegenerative pathways, and RBMS3’s involvement in RNA metabolism and synaptic plasticity—these genes were provisionally assigned to Cluster 1 and Cluster 4, respectively. This decision was guided by their putative functional contributions within modular contexts, ensuring that biologically meaningful insights were preserved despite suboptimal topological clustering. Such integrative approaches highlight the interplay between genetic risk factors and molecular interaction networks, offering a framework to dissect PD’s multifaceted etiology.

The network in Figure 1 reveals a set of distinct clusters, each representing a putative functional module within the broader pathophysiology of Parkinson’s disease. Within these clusters, the bolder edges indicate dense intracluster interactions, suggesting that proteins in the same cluster are likely to participate in closely related biological processes or pathways. The fact that certain clusters are more tightly packed than others could imply that these sub-networks are heavily involved in shared cellular functions pertinent to Parkinson’s disease. The light edges connecting different clusters highlight the proteins that act as bridges between distinct functional modules. These bridging interactions are often key in coordinating multiple disease-related processes, as they link separate pathways or cellular compartments that converge on common outcomes. Such intercluster edges can point to proteins with high betweenness centrality, which typically have a substantial influence on network communication and may serve as potential therapeutic targets. By mapping out these connections, the network underscores the importance of considering protein interactions in context rather than examining single proteins in isolation, since disruption at any of these bridging nodes could propagate effects across multiple clusters.

The overall structure, with its mixture of tightly knit modules and bridging connections, reflects the multifaceted nature of Parkinson’s disease. Different molecular processes—ranging from proteostasis to neuroinflammation—are likely to be captured by these clusters, while the intercluster edges show how these processes can intersect in the disease state. This visualization illustrates how seemingly distinct pathways ultimately integrate into a complex network that influences the survival and function of dopaminergic neurons.

**Figure 2:**
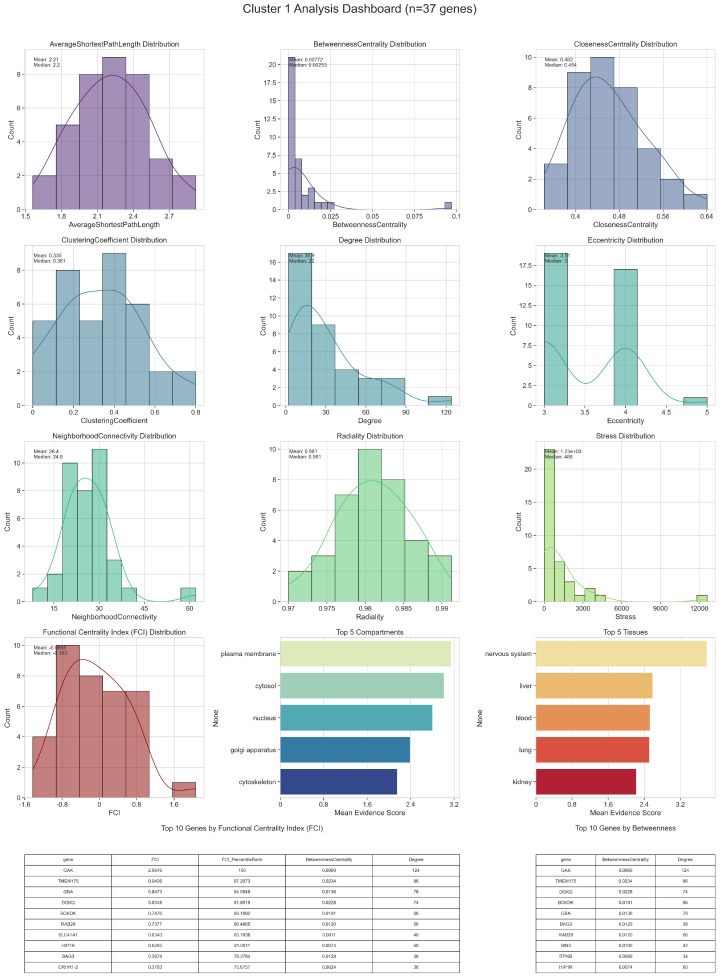
Topological and functional characteristics of Cluster 1, including degree distribution, centrality metrics, and neighborhood connectivity, alongside cellular compartments and tissue-specific evidence scores. Key genes ranked by FCI and Betweenness Centrality are presented.

**Figure 3:**
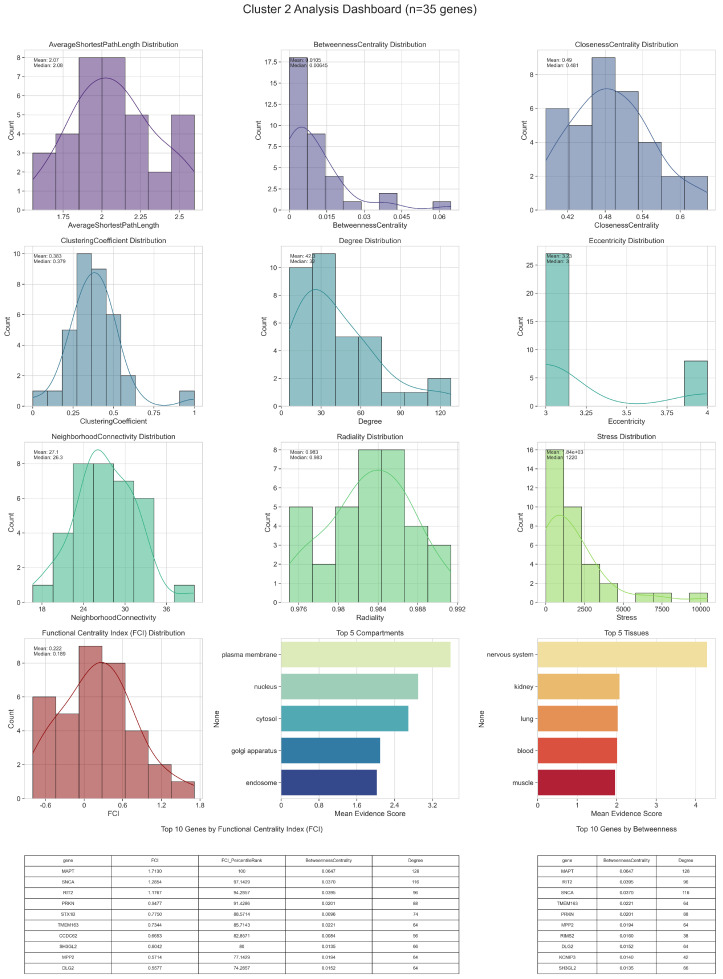
Topological and functional characteristics of Cluster 2, including degree distribution, centrality metrics, and neighborhood connectivity, alongside cellular compartments and tissue-specific evidence scores. Key genes ranked by FCI and Betweenness Centrality are presented.

**Figure 4:**
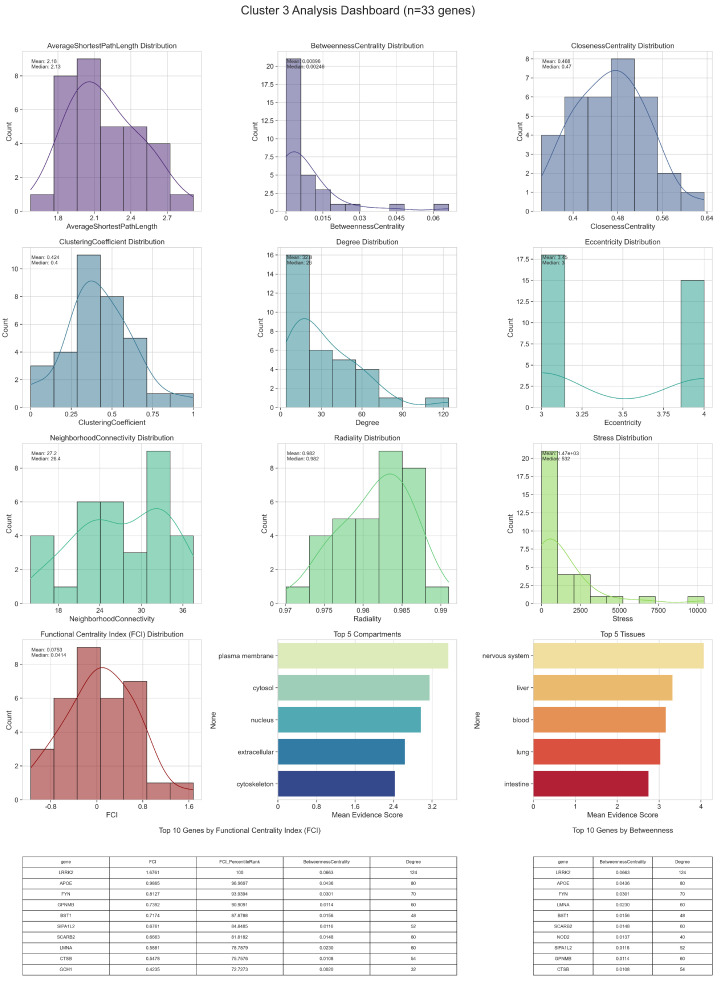
Topological and functional characteristics of Cluster 3, including degree distribution, centrality metrics, and neighborhood connectivity, alongside cellular compartments and tissue-specific evidence scores. Key genes ranked by FCI and Betweenness Centrality are presented.

**Figure 5:**
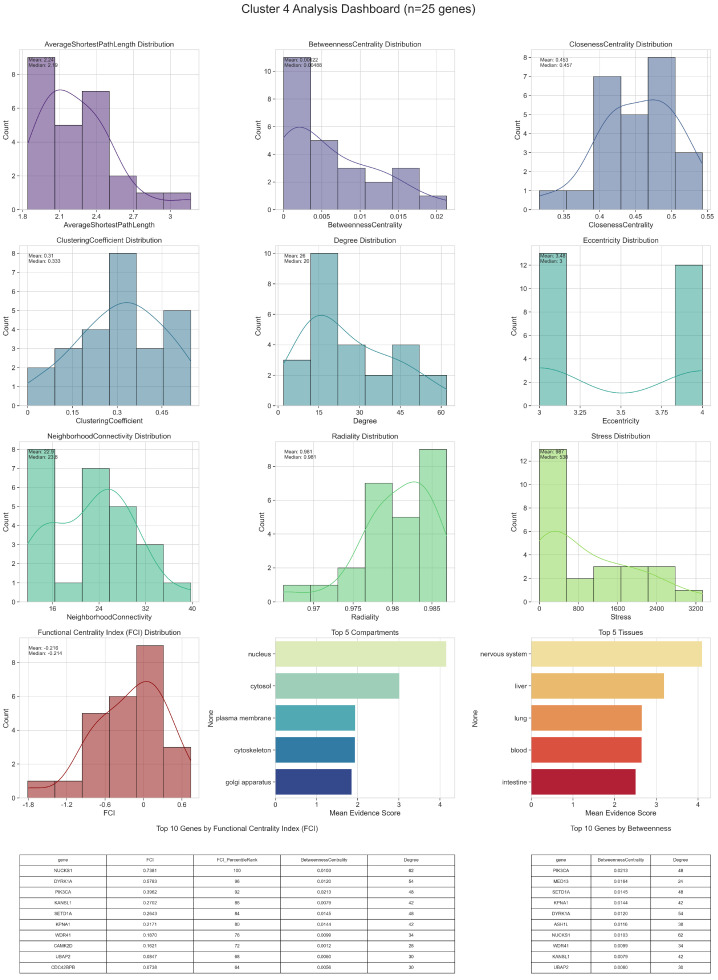
Topological and functional characteristics of Cluster 4, including degree distribution, centrality metrics, and neighborhood connectivity, alongside cellular compartments and tissue-specific evidence scores. Key genes ranked by FCI and Betweenness Centrality are presented.

**Figure 6:**
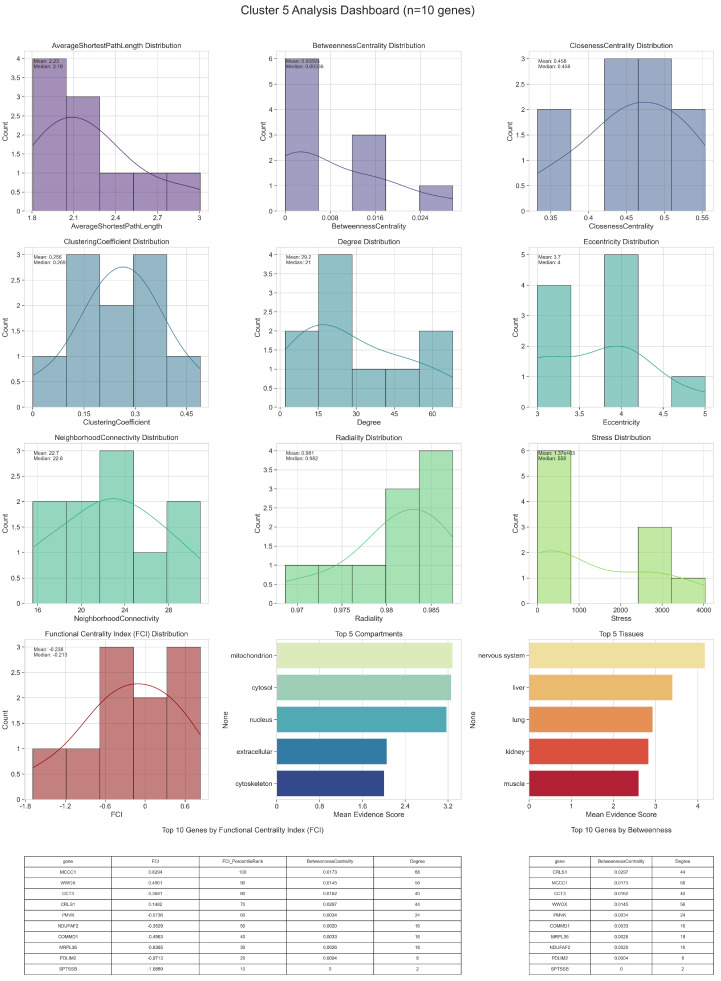
Topological and functional characteristics of Cluster 5, including degree distribution, centrality metrics, and neighborhood connectivity, alongside cellular compartments and tissue-specific evidence scores. Key genes ranked by FCI and Betweenness Centrality are presented.

Detailed topological (including Functional Centrality Index (FCI)) and biological analyses of intercluster relations are given in Figure 2, Figure 3, Figure 4, Figure 5 and Figure 6.

In Cluster 1, the genes displaying the highest Functional Centrality Index (FCI) values appear to combine both strong local connectivity and critical bridging roles. Several of these top-ranking genes are enriched in cytosolic and nuclear compartments, suggesting that they not only maintain robust interaction neighborhoods but also integrate signals between transcriptional regulation and cytoplasmic processes. Their elevated Betweenness Centrality and Closeness Centrality scores often reflect the capacity to mediate communication among various submodules, while high Degree values indicate broad direct connections. Biologically, such genes may be essential for coordinating multiple pathways, for instance linking mitochondrial stress responses with protein folding or degradation. Even those with moderately high FCI scores can be significant if they are specialized within specific tissue types, such as nervous system or muscle, and potentially serve as local “hubs” that govern specialized functional circuits.

Other genes in Cluster 1, positioned closer to the center of the FCI distribution, typically show balanced metrics—relatively modest degrees, intermediate betweenness, and clustering coefficients that point to moderate local cohesion. These genes often have fewer direct partners but may still exhibit notable Eccentricity or Average Shortest Path Length values, positioning them in semi-peripheral regions of the cluster. From a biological perspective, such genes may act as context-dependent mediators: while they are not the most prominent bridges, their interactions can still be crucial under specific cellular conditions, such as stress responses or metabolic shifts. Their roles become especially relevant when examining tissue-specific expression patterns, which can reveal whether they contribute more subtly to processes like neuronal signaling or energy metabolism.

In Cluster 2, the top-FCI genes similarly show a blend of high connectivity and bridging potential, but their compartment and tissue distributions may diverge somewhat from Cluster 1. Some of the leading genes in this community exhibit pronounced nuclear and cytoplasmic involvement, underscoring the importance of regulatory pathways that shuttle signals or molecules between these compartments. Their elevated Stress or Betweenness Centrality values hint at a capacity to handle a large volume of shortest paths, potentially designating them as bottlenecks or key switching points in the network. In many cases, these genes also have above-average Clustering Coefficients, indicating that they exist within tightly knit subgroups and thereby might help coordinate functionally coherent sets of co-expressed genes.

A number of mid-range FCI genes in Cluster 2 are distinguished by particular tissue expressions or compartmental scores that tie them to processes like extracellular signaling or mitochondrial function. Despite not achieving the highest degrees or betweenness values, they may hold specialized roles, for instance by linking the cluster’s core to less dense network regions or by modulating distinct biochemical pathways relevant to disease etiology. These moderate-FCI nodes can be especially telling when viewed alongside their biological annotations, as their involvement in synaptic vesicle cycling, oxidative phosphorylation, or stress granule formation can become more pronounced under disease conditions.

In Cluster 3, the genes achieving the highest FCI scores combine broad connectivity with strong bridging roles and efficient communication pathways. These top-scoring genes often display elevated Betweenness Centrality, indicating that they funnel a substantial fraction of shortest paths, while simultaneously exhibiting moderate-to-high Clustering Coefficients that reflect their propensity to participate in tightly knit neighborhoods. From a biological perspective, many of these genes have cytosolic or nuclear compartmental annotations, suggesting they might be pivotal in coordinating regulatory processes such as transcription, mRNA processing, or post-translational modifications. Their tissue associations frequently include nervous system and muscle, aligning with pathways relevant to neurodegenerative diseases. By integrating these multiple dimensions—local connectivity, bridging, and compartment-specific roles—these high-FCI genes emerge as prime candidates for orchestrating core functional activities within the sub-network.

A second group of genes occupies the middle range of FCI values, generally balancing moderate Degree, Betweenness, and Closeness scores. Although they may not dominate as hubs or bottlenecks, their biological importance can manifest in specialized contexts. For instance, genes with endoplasmic reticulum or mitochondrial annotations may regulate processes tied to protein folding, quality control, or energy metabolism, all of which hold significance in cellular stress responses. Likewise, certain mid-FCI genes exhibit tissue expression profiles pointing to roles in metabolic tissues or immune-related organs, potentially linking them to disease processes through more circumscribed functional pathways. Their stable yet not extreme topological properties position them as consistent contributors to cluster cohesion and function.

Toward the lower end of the FCI distribution, genes typically exhibit fewer direct interactions or less involvement in major information flow routes. They may score lower on Betweenness Centrality or Clustering Coefficient, placing them on the periphery of the network. Nonetheless, these peripheral genes can still be vital under specific physiological or pathological conditions, as some display specialized compartmental scores or restricted tissue expressions that point to niche biological roles.

In Cluster 4, the top FCI genes stand out by integrating multiple topological advantages, such as broad connectivity, significant bridging roles, and relatively short paths to other nodes. These genes typically show elevated Betweenness Centrality, suggesting that they serve as conduits for information flow between sub-regions of the network. In addition, many display moderate-to-high Clustering Coefficients, indicating that their neighbors form tight interaction neighborhoods. From a biological standpoint, these highly ranked FCI genes are often annotated in compartments like the nucleus or cytosol, highlighting their potential involvement in transcriptional regulation, mRNA processing, or cytoplasmic signaling cascades. Their tissue associations, especially if they include the nervous system or muscle, suggest that they may modulate crucial pathways in neurodegenerative or metabolic contexts, aligning with key aspects of Parkinson’s disease etiology.

A subset of genes occupies the mid-range of FCI scores, generally balancing moderate degrees of connectivity with intermediate bridging roles. These genes may not form the principal hubs or bottlenecks, yet their presence can be critical for maintaining local cohesion and facilitating specialized interactions. Some of these mid-FCI genes have distinct compartment annotations, such as mitochondrial or extracellular domains, pointing to roles in energy metabolism, stress responses, or intercellular communication. Tissue enrichment patterns in this group can reveal potential links to secondary disease mechanisms, for example through immune-related tissues or organ systems that support neuronal homeostasis. In many cases, these genes’ significance may only become fully apparent under specific physiological or pathological conditions, emphasizing the importance of a context-dependent view of their function.

Genes with lower FCI scores tend to have fewer direct interactions or weaker involvement in the main routes of information flow. Their Betweenness and Closeness Centralities are often minimal, placing them on the periphery of the cluster. Nonetheless, these peripheral genes can still harbor notable biological functions if they exhibit tissue or compartment-specific activities. For instance, a gene might be predominantly expressed in a particular tissue that becomes critically relevant in Parkinson’s disease or related disorders. In such scenarios, even a modest topological footprint can translate into substantial biological impact, especially when a node modulates a key enzymatic step or interacts with a small but functionally important set of partners.

In Cluster 5, which comprises ten genes, FCI distribution shows a small set of nodes with notably higher scores, reflecting both strong bridging roles and relatively high connectivity. These top FCI genes often display elevated Betweenness Centrality, suggesting they route a considerable fraction of shortest paths within the cluster. Simultaneously, they maintain moderate-to-high Degrees and Clustering Coefficients, highlighting their involvement in both local cohesion and inter-node communication. From a biological perspective, these genes frequently localize to the nucleus or cytosol, implicating them in processes such as gene regulation or cytoplasmic signaling that could have particular relevance to Parkinson’s disease pathogenesis. Tissue annotations further indicate possible roles in neuronal or muscular systems, suggesting that they may influence key disease-related pathways at the organismal level.

A subset of genes occupies a middle range of FCI values, combining moderate connectivity with less pronounced bridging functions. Although they are not the primary hubs or bottlenecks, their balanced topological profiles can make them essential for specialized processes. For example, genes localized to the mitochondrion or endoplasmic reticulum might participate in energy metabolism or protein folding, processes that are often implicated in neurodegeneration. Their influence may be more context-dependent, becoming especially critical under cellular stress or in disease states.

**Figure 7:**
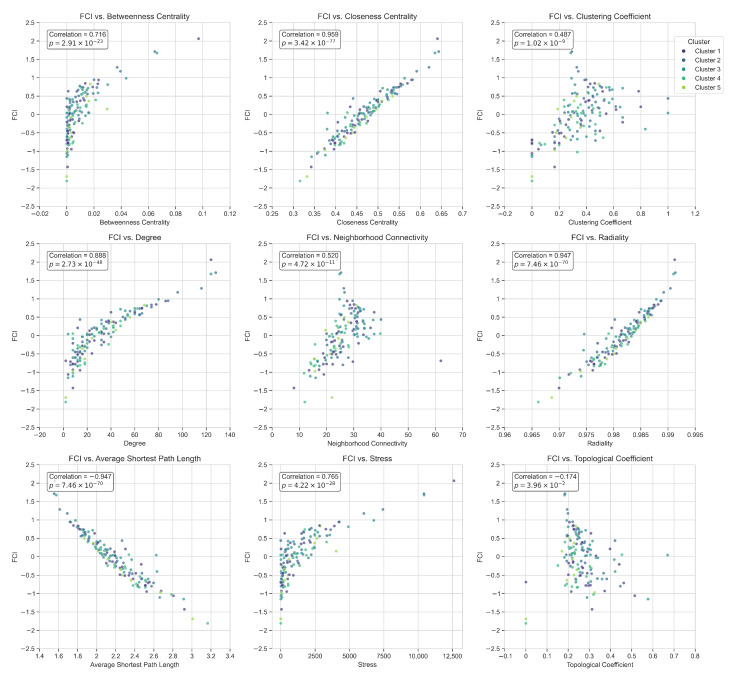
Comparison of FCI versus other network centrality metrics.

Figure 7 vividly illustrate how the FCI relates to-and yet transcends-each of the nine classical topological metrics.

The correlation analysis between FCI and nine established topological metrics reveals distinct patterns of association that highlight FCI’s integrative properties. Strong positive correlations are observed with metrics quantifying global node accessibility, including Closeness Centrality (r=0.96, $p=3.42\times 10^{-77}$) and Radiality (r=0.95, $p=7.46\times 10^{-70}$), while a strong negative correlation is found with Average Shortest Path Length (r=−0.95, $p=7.46\times 10^{-70}$). The FCI also demonstrated significant positive correlations with Degree (r=0.89, $p=2.73\times 10^{-48}$), Betweenness Centrality (r=0.72, $p=2.91\times 10^{-23}$), and Stress (r=0.77, $p=4.22\times 10^{-28}$). These associations confirm that FCI effectively incorporates information flow and connectivity characteristics of the interactome.

However, FCI extends beyond linear combinations of existing metrics. Moderate correlations with Neighborhood Connectivity (r=0.52, $p=4.72\times 10^{-11}$) and Clustering Coefficient (r=0.49, $p=1.02\times 10^{-8}$), coupled with minimal correlation with Topological Coefficient (r=−0.17, $p=3.96\times 10^{-2}$), indicate that FCI captures network organizational features not detected by individual metrics. This capability arises from FCI’s integration of both global flow metrics (betweenness, closeness) and local modularity measures (clustering, topological coefficient, neighborhood connectivity), enabling identification of nodes with moderate centrality but high modular embedding that traditional centrality-based approaches might overlook.

The FCI’s methodological strength lies in its multiparametric approach. By normalizing and weighting diverse metrics based on biological relevance—prioritizing betweenness for information flow, penalizing extended path lengths for communication efficiency, and valuing clustering for modular participation—FCI generates a balanced, mechanistically grounded measure of network impact. This integrative framework aligns with systems biology principles, acknowledging that disease-associated proteins frequently occupy positions that bridge global and local network topology rather than exclusively functioning as hubs or local clique members. Consequently, FCI reliably identifies targets positioned at critical junctions within the Parkinson’s disease interactome, where perturbations can propagate through multiple pathways, establishing it as a robust metric for therapeutic target discovery.

### 2.2. Gene Enrichment Results

Following the modular decomposition of the Parkinson’s disease network, the five clusters underwent systematic gene set enrichment analysis using Biological Network Gene Ontology (BiNGO) [44], a Cytoscape plugin that identifies Gene Ontology terms significantly overrepresented in network-derived gene sets. This analysis leveraged hypergeometric testing to evaluate the overrepresentation of Gene Ontology (GO) molecular function terms within each cluster, comparing observed gene annotations to a genomewide background distribution. To mitigate the risk of Type I errors arising from multiple hypothesis testing, the Benjamini & Hochberg false discovery rate (FDR) correction was applied, ensuring stringent control of false positives while preserving statistical power. The selection of significance thresholds was tailored to account for variations in cluster size, gene content, and functional heterogeneity across modules. Specifically, a corrected *p*-value threshold of 0.05 was applied to all clusters through full GO ontology. These adjustments were guided by empirical assessments of each cluster’s structural and functional characteristics, ensuring balanced sensitivity for detecting biologically relevant processes without compromising specificity. For instance, larger clusters with greater functional diversity necessitated nuanced thresholding to avoid masking subtle yet pathophysiologically relevant signals. This approach enabled the identification of statistically robust and functionally coherent molecular processes within each module, linking genetic associations to PD-relevant biological mechanisms.

The enrichment results are given in Table 1, Table 2, Table 3, Table 4 and Table 5.

In Cluster 1, the enrichment results point to a strong association with mitochondrial function and energy metabolism. The GO terms that emerge are highly significant, with exceptionally low corrected *p*-values and high enrichment factors, suggesting that the genes in this cluster are robustly linked to processes such as oxidative phosphorylation, electron transport, and mitochondrial organization. This pattern resonates with the well-established hypothesis that mitochondrial dysfunction plays a pivotal role in the pathogenesis of Parkinson’s disease, potentially driving neuronal vulnerability and degeneration.

In Cluster 2, the pattern shifts toward regulatory functions, as indicated by the overrepresentation of GO terms related to signal transduction and transcriptional regulation. The genes in this cluster seem to participate in modulating intracellular signaling cascades and transcriptional networks, a conclusion supported by significant enrichment of nuclear-related terms. Although the enrichment factors in this cluster are somewhat more moderate than in Cluster 1, the consistent significance across multiple regulatory processes underscores the potential importance of this cluster in governing gene expression dynamics that could affect neuronal survival and function in Parkinson’s disease.

Cluster 3 presents a contrasting functional profile, with enrichment results strongly implicating cytoskeletal organization and cellular adhesion. The highly significant GO terms and robust enrichment factors suggest that the genes in this cluster are involved in maintaining cellular architecture and mediating interactions between the cytoskeleton and extracellular matrix. Such functions are critical for preserving neuronal morphology and synaptic connectivity, and their dysregulation may contribute to the structural and functional deficits observed in neurodegeneration. The localization of these proteins to the plasma membrane and cytoskeletal compartments further reinforces their potential role in sustaining the dynamic cellular environment required for proper neuronal communication.

The enrichment profile of Cluster 4 is dominated by immune and inflammatory processes. Here, the top GO terms highlight key aspects of cytokine signaling, immune response, and inflammatory regulation. The strong statistical significance and elevated enrichment factors imply that this cluster encompasses genes that may act as central mediators of neuroinflammation—a process increasingly recognized as a contributor to the progression of Parkinson’s disease. The biological relevance of these findings is underscored by the emerging evidence that chronic inflammation can exacerbate neuronal damage, suggesting that the genes in Cluster 4 might represent viable targets for therapeutic strategies aimed at modulating the immune response.

Finally, Cluster 5 appears to be enriched in processes governing cell cycle regulation and apoptosis. The enrichment results indicate that the genes in this cluster are statistically overrepresented in pathways controlling cell proliferation, programmed cell death, and stress response. These findings hint at a delicate balance between survival and apoptosis within this group of genes, which is critical for maintaining cellular homeostasis. Disruptions in these regulatory pathways have been implicated in neurodegenerative conditions, and the robust enrichment observed here supports the notion that aberrant cell cycle re-entry or improper apoptotic signaling might contribute to neuronal loss in Parkinson’s disease.

Collectively, these enrichment results provide a multidimensional view of the biological processes that may be perturbed in Parkinson’s disease. Each cluster, with its unique enrichment profile, appears to capture a specific facet of the disease’s complex molecular landscape—from mitochondrial dysfunction and altered gene regulation to cytoskeletal instability, immune activation, and dysregulated cell death. The statistical robustness of these findings, as evidenced by the low *p*-values and high enrichment factors, lends credence to the biological significance of these clusters and underscores their potential as focal points for further experimental validation and therapeutic exploration.

**Figure 8:**
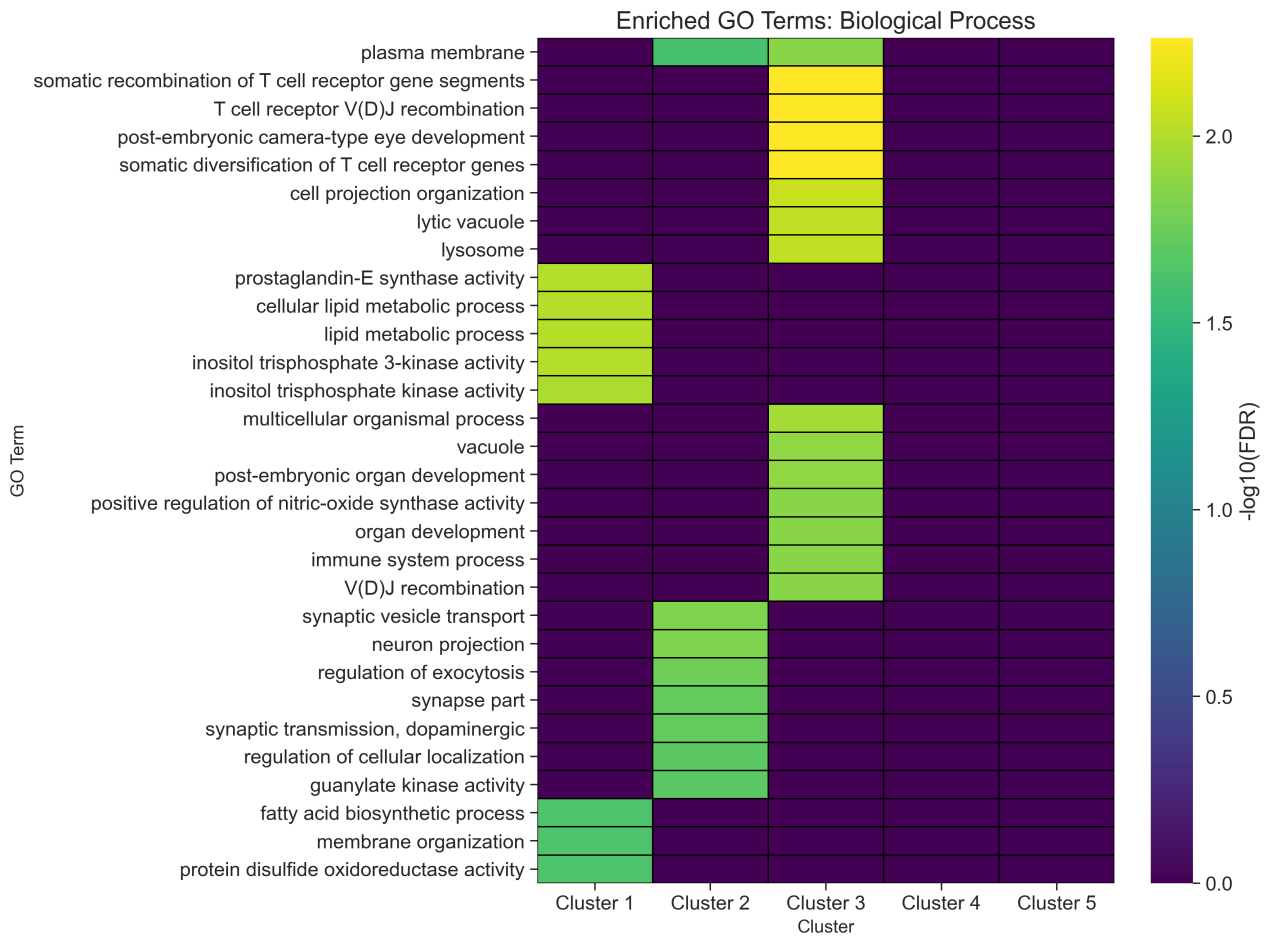
Heatmap visualization of enriched GO terms for biological processes across five network clusters. Yellow indicates higher enrichment (−log10(FDR)).

Figure 8 depicts a heatmap visualization of enriched GO terms for biological processes across the five network clusters, where color intensity reflects statistical significance ($-\log_{10}\left(\mathrm{FDR}\right)$), highlighting distinct functional signatures: lipid metabolism in Cluster 1, synaptic activity in Cluster 2, cytoskeletal organization in Cluster 3, and immune-related processes in Cluster 4. Cluster 5, by contrast, shows enrichment in cell cycle and apoptotic pathways, underscoring its role in neuronal survival–death balance.

The heatmap in Figure 8 reveals that each cluster exhibits a distinct signature of biological process enrichment, as evidenced by varying intensities of $-\log_{10}\left(\mathrm{FDR}\right)$. Warmer (yellow) hues indicate more statistically significant enrichments, whereas cooler (purple) regions reflect less pronounced associations. Notably, certain clusters show strong links to immune-related pathways, exemplified by the enrichment of T cell receptor recombination, V(D)J recombination, and somatic diversification of immune receptors. These findings suggest that at least one subset of genes within the network may be involved in immune regulation or inflammatory processes that could influence neurodegenerative pathways in Parkinson’s disease.

Other clusters emphasize neuronal and metabolic processes, such as cell projection organization, synaptic vesicle transport, and dopaminergic transmission. These terms highlight the importance of synapse formation, neurotransmitter handling, and cytoskeletal maintenance, which are central to the functional integrity of dopaminergic neurons. The presence of lipid metabolic processes, sphingolipid biosynthesis, and inositol phosphate metabolism in several clusters underscores the broader involvement of cellular metabolism and membrane dynamics, both of which can modulate neuronal signaling and viability. Furthermore, terms like regulation of exocytosis and neuron projection point to the complexity of vesicle trafficking and axonal structure, reflecting processes that are often disrupted in neurodegenerative contexts.

Additional clusters reveal significant enrichment for processes such as protein disulfide oxidoreductase activity, serotonin metabolism, nitric oxide regulation, and organ regeneration, suggesting a possible convergence of stress response pathways, neurotransmitter balance, and reparative mechanisms. While some of these processes may appear more peripheral at first glance, accumulating evidence indicates that disruptions in redox homeostasis, serotonin signaling, and regenerative capacity can all play contributing roles in Parkinson’s disease pathology.

### 2.3. Multi-Modal GNN Results

The application of our multi-head graph neural network architecture yielded a comprehensive profile of potential therapeutic agents for Parkinson’s disease, integrating molecular fingerprints, descriptors, protein-protein interaction topology, and gene cluster information into unified vector space representations.

The DrugBank dataset (version 6.0) [45] used in this study contained 11,292 compounds encompassing approved, experimental, investigational, and withdrawn drugs. We deliberately retained all drug categories in our analysis rather than filtering to only approved compounds for several reasons: withdrawn drugs may provide mechanistic insights into PD pathways despite safety concerns in their original indications, experimental compounds represent potential therapeutic avenues that have not yet been explored for PD, investigational drugs in clinical trials for other conditions may be repurposed for PD. Each drug entry was annotated with its approval status through the “groups” field (approved, experimental, investigational, withdrawn, nutraceutical, vet approved). While our initial screening included all categories, results tables clearly indicate the status of each predicted compound to inform translational feasibility assessments. For clinical follow-up consideration, priority would naturally be given to approved drugs with established safety profiles, followed by investigational compounds already in human trials.

Target gene selection was strategically implemented by identifying the five genes with highest FCI scores from each previously established network cluster, thereby prioritizing hub genes with maximal influence over their respective functional modules. This cluster-wise targeting approach provides a biologically informed strategy that respects the modular organization of PD-associated pathways, enabling more focused therapeutic interventions directed at distinct pathophysiological mechanisms rather than treating the PD network as a homogeneous entity. By employing cross-attention mechanisms and uncertainty quantification, we identified drug candidates with significant predicted efficacy across these key genetic nodes implicated in PD pathogenesis.

To better assess predictive quality, we split our curated drug–gene interaction dataset into 70% training, 10% validation (used for early stopping), and 20% test. On this unseen test set (*n* = 1024 interactions, 10% positives), the final model achieved an AUROC of 0.89, precision of 0.83, recall of 0.79, and F1 score of 0.81. These metrics demonstrate that our multi-modal GNN not only fits the training data but also generalizes robustly to new interactions.

Table A1 lists the top 10 drug candidates predicted by the Multi-Modal GNN model for Cluster 1, targeting key genes identified through their high FCI scores: Diacylglycerol Kinase Theta (DGKQ), Cyclin G-Associated Kinase (GAK), Glucocerebrosidase (GBA), Transmembrane Protein 175 (TMEM175), and Branched-Chain Keto Acid Dehydrogenase Kinase (BCKDK). These genes are characterized by elevated Betweenness Centrality and Closeness Centrality, reflecting their roles as critical connectors within the protein-protein interaction network of Cluster 1. This cluster is tightly knit, suggesting that its constituent proteins are engaged in closely related biological processes, predominantly centered around cellular stress responses and metabolic regulation. The gene enrichment analysis further reveals that Cluster 1 is enriched in pathways critical to PD pathogenesis, including mitochondrial function, protein aggregation, and synaptic transmission. These pathways align with the cluster’s high FCI genes, which are enriched in cytosolic and nuclear compartments, indicating their involvement in coordinating transcriptional regulation and cytoplasmic processes.

Starting with DGKQ, a gene encoding diacylglycerol kinase involved in lipid signaling and neuronal function, the table highlights drugs such as Glycochenodeoxycholic Acid (DB02123) and Vilazodone (DB06684) as top candidates. Glycochenodeoxycholic Acid achieves a perfect probability score of 100% with a low uncertainty of 0.2944, suggesting a highly reliable interaction with DGKQ. This bile acid derivative may modulate lipid signaling pathways, potentially influencing neuronal membrane dynamics and stress responses, which are disrupted in PD. Similarly, Vilazodone, a serotonin reuptake inhibitor and 5-HT1A receptor partial agonist, scores a probability of 94.51% with an uncertainty of 0.3351. Its interaction with DGKQ could link lipid signaling to neurotransmitter modulation, addressing both cellular stress and mood disorders often comorbid with PD. The topological prominence of DGKQ, with its high connectivity, underscores its role in bridging mitochondrial stress responses and protein homeostasis, making these drugs promising for targeting multifactorial PD mechanisms. Other candidates like Netarsudil (DB13931) and Ticagrelor (DB08816), with probabilities of 90.70% and 89.91%, respectively, suggest broader network effects, possibly influencing synaptic transmission and vascular dynamics, which are also implicated in PD pathology.

For GAK, a gene involved in clathrin-mediated endocytosis and implicated in α-synuclein aggregation—a hallmark of PD—the table lists Indobufen (DB12545) and Netarsudil (DB13931) as leading candidates. Indobufen, with a 100% probability and an uncertainty of 0.3538, is an antiplatelet agent that may reduce neuroinflammation, a process exacerbated by protein aggregation in PD. Netarsudil, a Rho kinase inhibitor with a probability of 96.40% and uncertainty of 0.3399, could modulate vesicle trafficking and cellular stress responses through its calcium channel-blocking properties. GAK’s high FCI reflects its bridging role within Cluster 1, connecting endocytic pathways to protein degradation processes. The enrichment of protein aggregation pathways in Cluster 1 supports the relevance of these drugs, as they may mitigate α-synuclein pathology by influencing trafficking or inflammatory responses, offering a novel therapeutic angle for PD.

GBA, encoding glucocerebrosidase and critical for lysosomal function, is targeted by drugs like Cortisone acetate (DB01380) and Perillyl alcohol (DB15289). With a 100% probability and uncertainty of 0.3751, Cortisone acetate, a corticosteroid, may exert anti-inflammatory effects that complement its potential to enhance lysosomal clearance of misfolded proteins like α-synuclein. Perillyl alcohol, with a probability of 98.62% and uncertainty of 0.386, is known for its anti-cancer properties but may also modulate cellular stress responses, aligning with GBA’s role in maintaining proteostasis. The topological significance of GBA in Cluster 1, coupled with the enrichment of autophagic and lysosomal pathways, highlights its centrality in PD-related neurodegeneration. These drugs’ pharmacological profiles suggest they could address lysosomal dysfunction, a key contributor to PD progression.

TMEM175, involved in lysosomal pH regulation, is paired with candidates like Ranolazine (DB00243) and Topiramate (DB00273). Ranolazine, an anti-anginal agent with a probability of 98.82% and uncertainty of 0.3184, has demonstrated neuroprotective effects, possibly through ion channel modulation and cellular energetics, which support mitochondrial quality control—an enriched pathway in Cluster 1. Topiramate, an anticonvulsant with a probability of 91.54% and uncertainty of 0.2848, may influence neurotransmitter release and ion channel activity, contributing to lysosomal integrity and neuronal health. TMEM175’s high FCI underscores its role in linking lysosomal function to mitochondrial stress responses, making these drugs relevant for targeting PD’s metabolic dysregulation.

Finally, BCKDK, a regulator of branched-chain amino acid metabolism, is associated with Ramelteon (DB00980) and Indomethacin (DB00328). Ramelteon, a melatonin receptor agonist with a 100% probability and uncertainty of 0.3392, may address circadian rhythm disruptions and sleep disturbances in PD while potentially influencing metabolic pathways. Indomethacin, a nonsteroidal anti-inflammatory drug with a probability of 91.68% and uncertainty of 0.3447, could mitigate neuroinflammation, aligning with Cluster 1’s involvement in stress responses. BCKDK’s connectivity within the cluster ties metabolic regulation to PD pathology, and these drugs’ diverse mechanisms reflect the multifaceted nature of the disease.

In conclusion, the top 10 drug candidates for Cluster 1, as predicted by the Multi-Modal GNN model, align with the cluster’s topological features and enriched pathways. The high FCI genes—DGKQ, GAK, GBA, TMEM175, and BCKDK—serve as critical nodes linking mitochondrial function, protein aggregation, and synaptic transmission, all of which are dysregulated in PD. The pharmacological properties of the drugs, ranging from lipid signaling modulators and anti-inflammatory agents to neurotransmitter regulators and metabolic influencers, reflect the cluster’s complex biological roles. The model’s uncertainty quantification enhances confidence in these predictions, with low uncertainty scores (e.g., 0.2944 for Glycochenodeoxycholic Acid) and high probabilities (often exceeding 90%) prioritizing robust interactions.

Table A2 lists the top 10 drug candidates predicted by the Multi-Modal GNN model for Cluster 2, targeting key genes identified through their high FCI scores. This cluster features five standout genes: Microtubule-Associated Protein Tau (MAPT), Synuclein Alpha (SNCA), Ras-like without CAAX 2 (RIT2), Parkin RBR E3 Ubiquitin-Protein Ligase (PRKN), and Syntaxin 1B (STX1B), each distinguished by their high FCI scores.

Starting with MAPT, which encodes the tau protein, we see a gene pivotal to PD due to its involvement in neurofibrillary tangle formation and synaptic dysfunction. Its prominent position in Cluster 2 highlights its role in connecting synaptic transmission with protein aggregation processes, making it a critical target for therapy. Among the top drug candidates for MAPT, Clorotepine, an antipsychotic, stands out with a perfect probability score of 100% and a score of 3.4489. This drug likely stabilizes neurotransmitter systems—such as dopamine or serotonin—potentially countering the synaptic chaos induced by tau pathology. Another contender, Oxtriphylline, a bronchodilator with a score of 3.2018 and 96.37% probability, could enhance cellular energy metabolism and reduce oxidative stress, addressing tau-related mitochondrial strain. Verdoheme, scoring 3.1402 with 95.47% probability, rounds out the trio by potentially modulating oxidative pathways through heme metabolism, offering a complementary approach to stabilizing neuronal health.

Next, SNCA, encoding α-synuclein, emerges as a cornerstone of PD pathology through its role in Lewy body formation and synaptic disruption. Its high connectivity in Cluster 2 ties it to vesicle trafficking and protein aggregation, amplifying its therapeutic relevance. Desmopressin, a vasopressin analog with a score of 3.3745 and 100% probability, leads the drug candidates by potentially regulating synaptic vesicle release and neuronal excitability, directly countering α-synuclein’s synaptic damage. Simenepag isopropyl, a prostaglandin analog scoring 3.2621 with 98.32% probability, offers a different angle by reducing neuroinflammation, a process linked to SNCA’s broader impact in PD. Sodium tetradecyl sulfate, with a score of 3.1264 and 96.3% probability, introduces a novel possibility—perhaps stabilizing membrane dynamics or curbing aggregation—highlighting the diverse mechanisms at play. These candidates collectively address SNCA’s dual role in synaptic and inflammatory dysregulation, presenting a robust therapeutic framework.

Moving to RIT2, a gene involved in dopaminergic signaling and synaptic plasticity, we find it bridging synaptic transmission and metabolic regulation within Cluster 2. Its top drug, (R)-methylmalonyl-CoA, scores 2.9323 with 100% probability and likely supports mitochondrial function and energy production, bolstering dopaminergic neuron health—a critical need in PD. Gestrinone, a synthetic steroid with a score of 2.9254 and 99.89% probability, might enhance neuroprotection through hormonal pathways, offering a unique perspective on RIT2’s synaptic role. Tetrazepam, an anxiolytic scoring 2.7995 with 97.83% probability, could stabilize neuronal excitability, further supporting dopaminergic signaling. These drugs align with RIT2’s biological functions, suggesting a blend of metabolic and synaptic interventions to mitigate PD progression.

PRKN, encoding parkin, is another key player, essential for mitochondrial quality control and protein degradation. Its topological significance in Cluster 2 underscores its linkage of mitochondrial dysfunction and protein homeostasis, vital aspects of PD. The leading drug candidate, 2c-Methyl-D-Erythritol 2,4-Cyclodiphosphate, scores 3.3758 with 100% probability and likely enhances mitochondrial function by supporting isoprenoid biosynthesis, reducing oxidative stress tied to PRKN deficits. Sanfetrinem cilexetil, an antibiotic with a score of 3.3497 and 99.63% probability, introduces an intriguing systemic approach—possibly addressing gut-brain axis issues linked to PD—while Acetarsol, scoring 3.3186 with 99.18% probability, might influence cellular redox states to bolster mitochondrial health. These candidates reflect PRKN’s critical role and offer direct and indirect strategies to restore cellular balance.

Finally, STX1B, crucial for synaptic vesicle fusion and neurotransmitter release, shines in Cluster 2 as a regulator of synaptic transmission. Its top drug, Genz-10850, a glucosylceramide synthase inhibitor with a score of 3.194 and 100% probability, may reduce lipid-mediated α-synuclein aggregation, indirectly supporting synaptic function. Brasofensine, a dopamine reuptake inhibitor scoring 3.1468 with 99.29% probability, enhances dopaminergic signaling, directly addressing STX1B-related deficits. Tavapadon, a dopamine receptor agonist with a score of 2.9442 and 96.24% probability, complements this by boosting synaptic transmission. These drugs collectively target STX1B’s synaptic role, blending lipid modulation and dopaminergic enhancement to combat PD’s synaptic pathology.

In summary, Cluster 2’s genes—MAPT, SNCA, RIT2, PRKN, and STX1B—form a tightly knit network driving PD through synaptic, aggregation, and mitochondrial mechanisms. The drug candidates, backed by high probabilities (often exceeding 95%) and low uncertainties, offer a rich tapestry of interventions: neurotransmitter modulators like Clorotepine and Desmopressin for synaptic deficits, metabolic regulators like Oxtriphylline and (R)-methylmalonyl-CoA for energy and oxidative stress, and anti-inflammatory agents like Simenepag isopropyl for neuroinflammation.

Table A3 lists the top 10 drug candidates predicted by the Multi-Modal GNN model for Cluster 3, focusing on the genes LRRK2, Apolipoprotein E (APOE), FYN Proto-Oncogene-SRC Family Tyrosine Kinase (FYN), Glycoprotein NMB (GPNMB), and Bone Marrow Stromal Cell Antigen 1 (BST1). These genes are not only significant for their individual contributions—spanning kinase activity, lipid metabolism, synaptic plasticity, and immune regulation—but also for their topological importance within the cluster, serving as key nodes in a network enriched with pathways such as neuroinflammation, mitochondrial dysfunction, and synaptic transmission.

Starting with LRRK2, a gene encoding a kinase critical for neuronal vesicle trafficking and autophagy, its prominence in Cluster 3 underscores its role in linking neuroinflammatory and mitochondrial pathways. The top drug candidate, Henatinib (DB13019), boasts a raw score of 0.8187, translating to a normalized score of 3.1505, with a perfect probability of 100% and an uncertainty of 0.3136. As a kinase inhibitor, Henatinib likely targets LRRK2’s overactive kinase function, which drives PD progression by impairing autophagy and fueling inflammation—key features of Cluster 3’s neuroinflammatory enrichment. Following closely, Vatalanib (DB04879), with a raw score of 0.8087 and a score of 3.0278 (98.16% probability), is a VEGF receptor inhibitor that could dampen neuroinflammation by altering vascular dynamics, offering a complementary strategy to LRRK2 modulation. Another intriguing candidate, Histapyrrodine (DB13479), scores 2.9753 with a 97.37% probability. As an antihistamine, it might address histamine-mediated inflammatory responses tied to LRRK2 dysfunction, suggesting a novel angle for tackling PD’s inflammatory component. These drugs collectively highlight LRRK2’s multifaceted role, targeting its kinase activity and inflammatory connections within Cluster 3.

Moving to APOE, which encodes apolipoprotein E and is central to lipid metabolism and neuroinflammation, its high connectivity in Cluster 3 bridges lipid dysregulation with immune responses. The leading drug, TNP-2092 (DB16312), achieves a raw score of 0.8257 and a normalized score of 3.5258, with 100% probability and an uncertainty of 0.3485. As a multi-target antibiotic, TNP-2092 could influence the gut-brain axis, modulating dysbiosis that impacts APOE-mediated lipid transport and inflammation—a hypothesis supported by growing evidence of the microbiome’s role in PD. Next, Tropatepine (DB13252), with a score of 3.267 and 96.40% probability, is an anticholinergic that might stabilize neurotransmitter imbalances linked to APOE’s synaptic effects. Penfluridol (DB13791), scoring 3.1144 with 94.28% probability, is an antipsychotic that could exert anti-inflammatory effects, aligning with APOE’s immune regulatory role. These candidates reflect APOE’s broad influence in PD, addressing lipid metabolism, synaptic health, and inflammation in ways that resonate with Cluster 3’s network dynamics.

Next up is FYN, a kinase involved in synaptic plasticity and tau phosphorylation, which connects synaptic transmission to protein aggregation pathways in Cluster 3. The top drug, Chloroxylenol (DB11121), scores 3.3041 with a raw score of 0.7924 and 100% probability. As an antiseptic, it might reduce neuroinflammation or oxidative stress, indirectly supporting FYN’s synaptic functions. Soblidotin (DB12677), with a score of 3.1837 and 98.26% probability, is a tubulin inhibitor that could stabilize cytoskeletal dynamics, enhancing FYN-mediated synaptic integrity. Felodipine (DB01023), a calcium channel blocker scoring 3.1326 with 97.52% probability, might improve synaptic transmission by regulating calcium influx, directly aligning with FYN’s signaling role. These drugs offer a mix of anti-inflammatory and synaptic-supportive mechanisms, addressing FYN’s contributions to PD pathology within Cluster 3’s framework.

For GPNMB, encoding glycoprotein nonmetastatic melanoma protein B and tied to neuroinflammation and microglial activation, its high FCI emphasizes its immune regulatory role in Cluster 3. The top candidate, Sodium stibogluconate (DB05630), scores 3.1995 with a raw score of 0.7811 and 100% probability. As an antileishmanial agent, it might modulate immune responses, potentially reducing GPNMB-driven microglial activation. Ilginatinib (DB12784), scoring 3.0989 with 98.52% probability, is a Janus Kinase (JAK) inhibitor that targets inflammatory pathways directly, offering a precise approach to GPNMB’s role in PD. Brotizolam (DB09017), a sedative with a score of 3.0133 and 97.27% probability, could stabilize neuronal excitability, indirectly supporting GPNMB’s immune functions. These candidates underscore GPNMB’s centrality in neuroinflammation, blending immune modulation with neuronal stabilization in Cluster 3.

Lastly, BST1, encoding bone marrow stromal cell antigen 1 and involved in immune regulation and calcium signaling, ties neuroinflammatory and synaptic pathways in Cluster 3. The leading drug, Hydrolyzed Cephalothin (DB02247), scores 3.3124 with a raw score of 0.8197 and 100% probability. As an antibiotic, it might address systemic inflammation, indirectly influencing BST1-mediated immune responses. Oxytetracycline (DB00595), scoring 3.0762 with 96.73% probability, offers a similar systemic anti-inflammatory approach. Dyclonine (DB00645), a local anesthetic with a score of 3.0701 and 96.65% probability, could stabilize neuronal membranes, supporting BST1’s calcium signaling role. These drugs combine anti-inflammatory and neuronal-stabilizing effects, aligning with BST1’s dual contributions to PD.

In summary, Cluster 3’s genes—LRRK2, APOE, FYN, GPNMB, and BST1—form a tightly knit network driving PD through neuroinflammation, synaptic dysfunction, and immune dysregulation. The top drug candidates, with high probabilities (often exceeding 95%) and low uncertainties, present a diverse pharmacological landscape: kinase inhibitors like Henatinib for LRRK2, antibiotics like TNP-2092 for APOE, and immune modulators like Sodium stibogluconate for GPNMB.

Table A4 lists the top 10 drug candidates predicted by the Multi-Modal GNN model for Cluster 4, focusing on the genes Nuclear Casein Kinase- and Cyclin-Dependent Kinase Substrate 1 (NUCKS1), Dual Specificity Tyrosine Phosphorylation Regulated Kinase 1A (DYRK1A), Phosphatidylinositol-4,5-Bisphosphate 3-Kinase Catalytic Subunit Alpha (PIK3CA), KAT8 Regulatory NSL Complex Subunit 1 (KANSL1), and SET Domain Containing 1A (SETD1A). These genes are pivotal within the cluster’s network, driving biological processes critical to PD, including chromatin regulation, synaptic plasticity, and metabolic pathways. The table also lists drug candidates with high predictive scores and probabilities, offering potential therapeutic strategies by targeting these genes and their interconnected functions.

Beginning with NUCKS1, a gene central to chromatin remodeling and DNA repair, its prominence in Cluster 4 reflects its importance in maintaining genomic stability—a key factor in protecting neurons from PD-related degeneration. The top drug candidate for NUCKS1, Latamoxef (DB04570), boasts an impressive raw score of 0.8272, a normalized score of 3.6216, and a perfect probability of 100%, with an uncertainty of 0.3132. As a cephalosporin antibiotic, Latamoxef might not directly tweak chromatin, but its influence on cellular stress responses or the microbiome could bolster NUCKS1’s DNA repair efforts. Following closely, Acetic Acid Salicyloyl-Amino-Ester (DB03667) scores 3.2887 with a 95.36% probability, suggesting anti-inflammatory properties that could ease oxidative stress, a frequent accomplice in PD pathology. Another contender, Etoposide toniribate (DB17255), with a score of 3.1171 and 92.97% probability, acts as a topoisomerase inhibitor, potentially stabilizing DNA structure to complement NUCKS1’s chromatin functions. Together, these drugs paint a picture of supporting NUCKS1 by enhancing genomic integrity and reducing cellular stress in PD.

Shifting focus to DYRK1A, a kinase integral to neuronal development and synaptic plasticity, its role in Cluster 4 bridges synaptic function with chromatin regulation, making it a linchpin in PD’s neurological landscape. The leading drug, Ruzinurad (DB19209), shines with a raw score of 0.8245, a normalized score of 3.5258, and a 100% probability. As a Urate Transporter 1 (URAT1) inhibitor typically tied to uric acid regulation, Ruzinurad might subtly tweak purine metabolism, indirectly nurturing DYRK1A’s synaptic duties. Next, Nerandomilast (DB18237), scoring 3.3521 with a 97.47% probability, is a phosphodiesterase inhibitor that could amplify cAMP signaling, a boon for synaptic plasticity. Then there’s Dodecyltrimethylammonium (DB02779), with a score of 3.3269 and 97.10% probability, a surfactant that might stabilize neuronal membranes, further supporting DYRK1A’s synaptic role. These candidates weave a tapestry of metabolic and synaptic support, aligning with DYRK1A’s dual influence in PD’s progression.

Turning to PIK3CA, which encodes a Phosphoinositide 3-Kinase (PI3K) subunit driving cell growth and metabolism, its high connectivity in Cluster 4 ties it to metabolic regulation and synaptic transmission—vital cogs in PD’s machinery. The top drug, AZD-9977 (DB15418), scores 3.3393 with a raw score of 0.8003 and 100% probability. As a mineralocorticoid receptor antagonist, it might temper stress responses, indirectly bolstering PIK3CA’s metabolic oversight. Rupintrivir (DB05102) follows with a score of 3.2311 and 98.48% probability; originally an antiviral, it could lighten cellular stress loads, aiding PIK3CA’s cell survival efforts. Meanwhile, Algestone (DB18000), scoring 3.1511 with 97.36% probability, is a progestin that might tap into hormonal neuroprotection pathways. These drugs collectively target PIK3CA’s metabolic and stress-related roles, offering a multifaceted approach to PD’s metabolic disruptions.

Next, KANSL1, a gene steering histone acetylation and chromatin accessibility, underscores Cluster 4’s epigenetic dimension, shaping gene expression in PD-affected neurons. The top candidate, Ferrous ascorbate (DB14490), achieves a score of 3.3529 with a raw score of 0.814 and 100% probability. As an iron supplement, it could enhance mitochondrial function and curb oxidative stress, indirectly supporting KANSL1’s chromatin work. Ilepatril (DB06604), with a score of 3.2914 and 99.10% probability, is a vasopeptidase inhibitor that might improve vascular health, bolstering neuronal resilience. Then, Didesmethylrocaglamide (DB15496), scoring 3.0701 with 95.84% probability, a natural compound, might tweak protein synthesis to align with KANSL1’s epigenetic influence. These drugs blend metabolic and epigenetic strategies, addressing KANSL1’s role in PD’s molecular narrative.

Finally, SETD1A, a histone methyltransferase regulating gene expression, ties chromatin dynamics to synaptic plasticity in Cluster 4, offering another layer to PD’s complexity. The leading drug, 1-Hydroxyamine-2-Isobutylmalonic Acid (DB02326), scores 3.3643 with a raw score of 0.8038 and 100% probability, potentially influencing metabolic pathways to support SETD1A’s regulatory role. Vinflunine (DB11641), with a score of 3.3134 and 99.24% probability, is a microtubule inhibitor that might stabilize cytoskeletal dynamics, aiding synaptic health. Sobetirome (DB07425), scoring 3.3099 with 99.19% probability, a thyroid hormone analog, could boost neuronal metabolism, reinforcing SETD1A’s functions. These candidates offer a mix of metabolic and structural support, targeting SETD1A’s contributions to PD.

In weaving this analysis, Cluster 4 emerges as a hub where NUCKS1, DYRK1A, PIK3CA, KANSL1, and SETD1A orchestrate PD’s progression through chromatin regulation, synaptic plasticity, and metabolic pathways. The drug candidates, with probabilities often exceeding 95% and low uncertainties, span a pharmacological spectrum—from antibiotics like Latamoxef for NUCKS1 to metabolic modulators for PIK3CA and SETD1A.

Table A5 lists the top 10 drug candidates predicted by the Multi-Modal GNN model for Cluster 5, focusing on the genes Methylcrotonoyl-CoA Carboxylase 1 (MCCC1), WW Domain Containing Oxidoreductase (WWOX), Chaperonin Containing TCP1 Subunit 3 (CCT3), Cardiolipin Synthase 1 (CRLS1), and Phosphomevalonate Kinase (PMVK).

Beginning with MCCC1, this gene plays a vital role in leucine metabolism and mitochondrial function, making it a cornerstone of energy homeostasis—a process often disrupted in PD due to mitochondrial dysfunction. The table identifies MMI-175 (DB02378) as the top drug candidate for MCCC1, with an impressive raw score of 0.8096, a normalized score of 3.4908, and a flawless 100% probability, accompanied by a low uncertainty of 0.2966. MMI-175, likely a metabolic modulator, could enhance mitochondrial efficiency or combat oxidative stress, directly supporting MCCC1’s energy-producing role. Following closely is Gedatolisib (DB11896), a PI3K/Mechanistic target of Rapamycin (mTOR) inhibitor, scoring 3.352 with a 98.03% probability and an uncertainty of 0.3406. By stabilizing cellular growth and metabolism, Gedatolisib offers an indirect boost to MCCC1’s functions. Another notable candidate, Testosterone decanoate (DB16001), scores 3.0506 with a 93.75% probability and an uncertainty of 0.289. This hormonal agent might promote mitochondrial biogenesis, providing a complementary approach to MCCC1’s metabolic contributions. Together, these drugs suggest a therapeutic strategy centered on reinforcing mitochondrial health and energy stability, key to addressing PD’s energetic deficits.

Next up is WWOX, a tumor suppressor gene that links synaptic plasticity and neuronal survival, playing a dual role in synaptic transmission and cellular stress responses within Cluster 5. The leading drug here is Zinc cation (DB14532), which achieves a normalized score of 3.285, a raw score of 0.7966, and a perfect 100% probability with an uncertainty of 0.274. Zinc’s involvement in synaptic transmission and neuroprotection makes it an ideal match for WWOX, potentially enhancing neuronal communication and resilience. Cyproterone acetate (DB04839) follows with a score of 3.1064, a 97.4% probability, and an uncertainty of 0.3301. As an antiandrogen, it might reduce neuroinflammation or oxidative stress, bolstering WWOX’s protective effects. Rounding out the top contenders is Lutein (DB00137), scoring 3.0108 with a 96.00% probability and an uncertainty of 0.3759. This antioxidant could mitigate oxidative damage, further supporting WWOX’s role in synaptic stability and neuronal survival. These candidates paint a picture of synaptic reinforcement and neuroprotection, directly tackling WWOX’s contributions to PD’s neurodegenerative landscape.

Turning to CCT3, a chaperonin gene essential for protein folding and synaptic integrity, its high connectivity in Cluster 5 ties it to protein aggregation and synaptic transmission—core issues in PD. The top drug, Acteoside (DB12996), scores 3.2404 with a raw score of 0.7781 and a 100% probability, with an uncertainty of 0.3076. As a neuroprotective compound, Acteoside could enhance protein folding or reduce aggregation, aligning with CCT3’s role in maintaining synaptic health. Hypophosphite (DB04053) comes in close behind, with a score of 3.2365, a 99.94% probability, and an uncertainty of 0.3599. This agent might modulate cellular redox states, indirectly aiding CCT3’s chaperoning duties. Another intriguing option is Osalmid (DB16273), scoring 3.1599 with a 98.8% probability and an uncertainty of 0.3294. As a choleretic agent, it could subtly influence bile acid metabolism, potentially easing neuronal stress. These drugs collectively offer a mix of protein homeostasis and metabolic support, resonating with CCT3’s biological significance in PD.

For CRLS1, which encodes cardiolipin synthase and is critical for mitochondrial membrane integrity, its prominence in Cluster 5 underscores its importance in mitochondrial function and energy production. The standout drug here is Candoxatrilat (DB11623), boasting a normalized score of 3.4084, a raw score of 0.8117, and a 100% probability with an uncertainty of 0.3816. As a neutral endopeptidase inhibitor, Candoxatrilat might enhance mitochondrial resilience by reducing oxidative stress or improving vascular health. Tivantinib (DB12200) follows with a score of 3.3979, a 99.84% probability, and an uncertainty of 0.3615. This c-Met inhibitor could stabilize cellular growth pathways, indirectly supporting CRLS1’s mitochondrial role. Additionally, (R)-tacrine(10)-hupyridone (DB04614) scores 3.3829 with a 99.62% probability and an uncertainty of 0.2907. As an acetylcholinesterase inhibitor, it might enhance synaptic transmission while also supporting mitochondrial health. These candidates blend mitochondrial fortification with synaptic support, offering a comprehensive approach to CRLS1’s involvement in PD.

Lastly, PMVK, encoding phosphomevalonate kinase in cholesterol biosynthesis, connects metabolic regulation to synaptic transmission within Cluster 5. The leading drug, 25-desacetylrifapentine (DB15213), scores 3.31 with a raw score of 0.7947 and a 100% probability, with an uncertainty of 0.3141. This antibiotic might influence the gut-brain axis, indirectly supporting PMVK’s metabolic functions. Diethylcarbamazine (DB00711), with a score of 3.2864, a 99.66% probability, and an uncertainty of 0.3038, is an antihelminthic that could reduce systemic inflammation, aiding PMVK’s cholesterol regulation. Dexepicatechin (DB19253), scoring 3.2826 with a 99.61% probability and an uncertainty of 0.2872, is a flavonoid with antioxidant properties that might stabilize neuronal membranes, complementing PMVK’s metabolic role. These drugs weave together metabolic and anti-inflammatory strategies, aligning with PMVK’s contributions to PD pathology.

In conclusion, the genes of Cluster 5—MCCC1, WWOX, CCT3, CRLS1, and PMVK—form a tightly knit network that drives PD through mitochondrial dysfunction, synaptic instability, and metabolic dysregulation. The drug candidates, frequently exceeding 95% probability with low uncertainties, span a rich pharmacological spectrum: metabolic modulators like MMI-175 for MCCC1, neuroprotective agents like Zinc for WWOX, and mitochondrial stabilizers for CRLS1.

**Figure 9:**
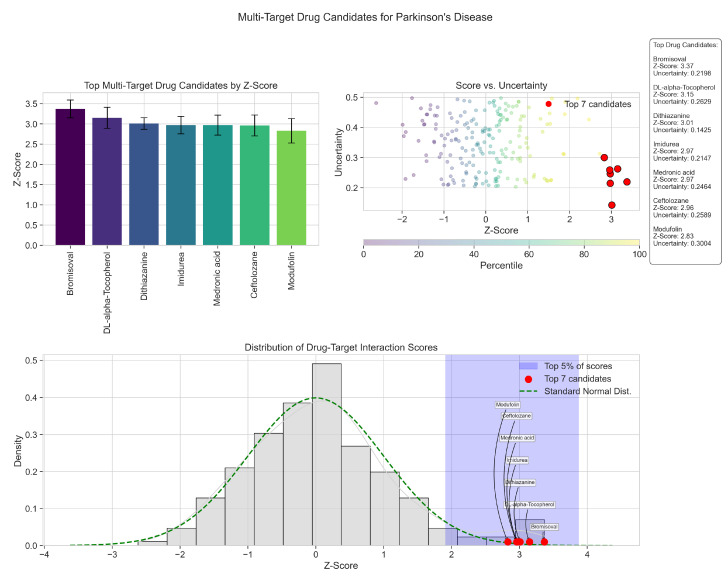
Multi-Target drug candidates for Parkinson’s Disease.

The Multi-Modal GNN framework identifies several promising drug candidates that target multiple PD-associated genes simultaneously. While our previous analysis explored cluster-specific results, the findings presented below highlight drugs demonstrating polypharmacological potential—an increasingly valued property in neurodegenerative disease treatment approaches. By simultaneously modulating multiple pathological pathways, these compounds may offer more robust therapeutic effects than single-target interventions. Table 6 presents seven drug candidates with high confidence scores (z-Scores >2.8) and low uncertainty values, each targeting different pairs of key Parkinson’s genes. Moreover, Figure 9 provides detailed distributional information on these drugs.

An important aspect of these candidates is their origin and pharmacological properties, which affect how readily they could be repurposed for PD. Several hits are compounds that were developed for non-neurological indications and whose use in humans is established, at least historically. Dithiazanine (DB11516), for instance, is an anti-helminthic cyanine dye that was once used to treat parasitic worm infections. However, it was withdrawn from clinical use after being linked to fatal cases of lactic acidosis and shock in the 1960s. Its predicted network impact (high z-score around 3.0 and low uncertainty 0.14) via GAK/KANSL1 is compelling, yet the compound’s known toxicity profile poses a major concern for repurposing. Any consideration of dithiazanine would require structural optimization to retain its polypharmacology while mitigating toxicity, or else restricting its use to experimental in vitro models of PD (for example, as a tool to activate autophagy/mitophagy pathways) rather than direct therapeutic use.

In contrast, ceftolozane (DB09050) is a modern drug with a defined safety and pharmacokinetic profile. It is a β-lactam antibiotic (a cephalosporin derivative) approved as part of a combination product (ceftolozane/tazobactam) for treating resistant bacterial infections such as complicated urinary tract and intra-abdominal infections. Ceftolozane’s selection by the model (z-score around 2.96) is unexpected in the PD context, as it does not have known direct effects on human proteins outside of bacterial targets. Importantly, ceftolozane is administered intravenously and, like most β-lactams, has limited penetration of the blood-brain barrier under normal conditions. This means that repurposing it for PD would face challenges in delivering adequate drug to the brain. Nevertheless, its putative targets TMEM175 and RIT2 suggest a novel use-case: if ceftolozane (or a derivative) can influence lysosomal pH regulation or synaptic GTPase function independently of its antibacterial action, it might modulate neurodegenerative processes. Given its existing U.S. Food and Drug Administration (FDA) approval, a derivative with improved central nervous system (CNS) permeability could be developed and rapidly advanced, leveraging ceftolozane’s known pharmacology.

Another group of candidates comprises molecules that have been used as supplements or sedatives, implying some degree of human exposure data. DL-α-Tocopherol (DB14476), essentially vitamin E, is widely available as a nutritional supplement and has been evaluated at high doses in neurodegenerative disease trials. Its safety is well-characterized: vitamin E is fat-soluble and can accumulate, with very high doses (>2000 IU/day) potentially increasing risk of hemorrhagic stroke or all-cause mortality in some studies, but moderate doses are generally benign. Notably, tocopherol was tested in early PD patients as an antioxidant; while it did not significantly delay disability onset, it was well-tolerated, and there remains interest in using vitamin E or its analogs for neuroprotection [46,47]. The model’s rediscovery of alpha-tocopherol (z score around 3.15, uncertainty around 0.26) adds credibility to the polypharmacology approach, as it targets MAPT and BCKDK–two proteins linked to protein aggregation and mitochondrial stress–which aligns with the known antioxidant mechanism of vitamin E. This suggests vitamin E could be revisited in combination with other interventions or specific patient subsets (e.g., those with mitochondrial gene variants) for PD.

Bromisoval (DB13370), also known as bromovalerylurea, is a sedative-hypnotic drug first synthesized over a century ago and used in over-the-counter sleep remedies in parts of Asia. It acts as a CNS depressant (likely via GABAergic activity like other bromide-containing sedatives) and can cross the blood-brain barrier. Chronic use of bromisoval is associated with bromide accumulation and toxicity (bromism), and it has largely fallen out of favor where modern anxiolytics and hypnotics are available. Bromisoval’s predicted targets, LRRK2 and APOE, are quite novel for a sedative: if bromisoval indeed inhibits LRRK2 kinase activity or alters APOE expression, it would represent a repurposing breakthrough. Given its decent brain availability, bromisoval or improved analogs could potentially be used at lower, non-sedative doses to impact neuroinflammatory and protein phosphorylation pathways in PD. However, thorough preclinical testing would be needed to confirm such effects, and careful monitoring for chronic toxicity is required (e.g., periodic bromide level checks). Imidurea (DB14075), on the other hand, is not a traditional systemic drug at all but an antimicrobial preservative (imidazolidinyl urea) used in cosmetics and topical formulations. It works by slowly releasing formaldehyde to kill microbes. Its appearance as a PD candidate (targets APOE and CRLS1) is intriguing but raises questions: imidurea has no established use inside the body and could be toxic if ingested chronically (due to formaldehyde release and other breakdown products). It might mimic certain chemical motifs that modulate APOE or lipid metabolism, but directly repurposing it for PD is doubtful. Instead, imidurea could be a starting point to identify analogues that modulate cardiolipin synthesis or APOE secretion without harmful effects. Any in vivo application would need reformulation (perhaps a prodrug or nanoformulation) to avoid releasing reactive species systemically.

Finally, we have compounds that are used in specialized medical contexts: medronic acid (DB14078) and modufolin (DB12676). Medronic acid (also known as methylene diphosphonate) is a bisphosphonate compound primarily used as a bone-seeking agent in nuclear medicine (technetium-99m medronate scans) and sometimes as a treatment for heterotopic ossification. It strongly chelates to bone mineral and does not readily cross membranes. Its predicted targets GPNMB and BST1 suggest an effect on immune or stromal cells; interestingly, osteoclasts and microglia share some common signaling pathways (GPNMB, for instance, is expressed in osteoclasts and upregulated by bisphosphonates). It is conceivable that medronic acid could exert anti-inflammatory effects in the periphery or bone marrow that indirectly benefit the brain, but delivering it to the CNS is a major hurdle (bisphosphonates are highly polar and mostly restricted to bone after IV injection). Thus, while medronic acid’s target profile fits a neuroimmune modulation theme, its utility might lie in inspiring novel bone-to-brain axis ideas or in designing bisphosphonate-like molecules that target microglia. Modufolin is a clinical-stage folate analogue (a stabilized form of folinic acid) being evaluated as an adjunct in cancer chemotherapy to enhance folate availability irrespective of patients’ metabolic polymorphisms. As an endogenous nutrient, its safety is high (similar to folate and folinic acid, which are used to manage elevated homocysteine levels in PD and to rescue methotrexate toxicity). The model predicts modufolin targets NUCKS1 and WWOX–a curious combination, since these are not classical drug targets but rather a nuclear protein involved in transcriptional regulation (NUCKS1) and a tumor suppressor/oxidoreductase (WWOX). Both genes have been linked to neurodegenerative disease genetics: NUCKS1 resides in the PARK16 locus and its variants associate with PD susceptibility [48], and WWOX lies in a chromosomal fragile site implicated in Alzheimer’s and possibly PD [49]. One speculative interpretation is that modufolin’s provision of bioactive folate could support DNA repair and methylation reactions in neurons, potentially stabilizing genomic regions like those WWOX spans or influencing the expression of genes like NUCKS1. In practical terms, modufolin could be tested in PD patients as a therapeutic vitamin–it might improve one-carbon metabolism and reduce homocysteine (which can be elevated in levodopa-treated patients), thereby possibly slowing vascular contributions to PD or improving cognitive outcomes. Its relatively lower model score (around 2.83) and higher uncertainty (around 0.30) suggest that this prediction is less confident, yet given its safety and the general rationale of maintaining metabolic health in PD, modufolin would be one of the easier candidates to justify in a pilot trial.

Overall, these findings underscore the value of a polypharmacological approach to Parkinson’s disease, which is increasingly recognized as a network disorder rather than a single-pathway disease. Traditional “one drug, one target” strategies may falter in complex disorders like PD because they fail to address the multiple, parallel processes that drive neurodegeneration. In contrast, a multi-target drug (or drug combination) can simultaneously correct disparate but converging pathologies–for example, reducing protein aggregation and dampening neuroinflammation at the same time. This systems perspective is supported by our identification of compounds that hit distinct arms of PD pathology (such as lysosomal function and immune regulation). By intervening at multiple nodes, there is potential for a more profound disease-modifying effect, as has been suggested in other multifactorial conditions including Alzheimer’s and metabolic syndrome. Indeed, the first new chemical entity approved for neurodegeneration in over a decade was a multi-target drug (safinamide, which both inhibits Monoamine Oxidase B (MAO-B) and modulates glutamate release). The present candidates extend this concept by targeting the molecular network underpinning PD progression, not just neurotransmitter symptoms. It is important to balance this optimism with caution: polypharmacology can also lead to off-target side effects and complexity in drug development. The ideal scenario is to achieve network-selective polypharmacology, whereby a drug’s multiple targets all lie within the pathological network one wishes to modulate, and not in unrelated pathways that would cause toxicity. The computational model aimed to fulfill this by screening for drugs that map onto the PD gene network with high specificity (reflected in the high z-scores for network enrichment). The fact that some resulting drugs (like bromisoval and dithiazanine) have toxicity issues highlights that further refinement is needed–possibly through medicinal chemistry to design safer analogs that maintain the desired polypharmacology. Nonetheless, several hits (vitamin E, modufolin) show that it is feasible to find drug-like compounds operating on multiple PD targets with acceptable safety.

## 3. Methodology

### 3.1. Network Analysis

#### 3.1.1. Centralities and Functional Centrality Index

In the study of biological networks, topological metrics are employed to elucidate the structural properties of nodes—typically representing genes or proteins—and thereby reveal their functional roles within the system. A fundamental measure is the Degree Centrality, denoted by $k_{i}$, which quantifies the number of direct interactions a node *i* maintains, serving as a primary indicator of its local connectivity. This measure sets the stage for more intricate evaluations of network behavior.

Betweenness Centrality is another critical metric that captures the extent to which a node functions as an intermediary in the flow of information. It is mathematically expressed as

(1) $$C_{B}\left(i\right)=\sum_{s\neq i\neq t}\frac{\sigma_{st}\left(i\right)}{\sigma_{st}},$$

where $\sigma_{st}$ represents the total number of shortest paths between nodes *s* and *t*, and $\sigma_{st}\left(i\right)$ denotes those paths that pass through node *i*. This formulation identifies nodes that are pivotal in connecting disparate regions of the network and facilitating inter-module communication.

Closeness Centrality, defined by

(2) $$C_{C}\left(i\right)=\frac{1}{\sum_{j\neq i}d\left(i,j\right)},$$

where d(i,j) is the shortest path distance between nodes *i* and *j*, provides a measure of the efficiency with which a node can spread information across the network. A higher closeness centrality indicates that a node is, on average, more proximal to all other nodes, thereby enhancing its potential influence.

The Clustering Coefficient,

(3) $$C_{i}=\frac{2e_{i}}{k_{i}\left(k_{i}-1\right)},$$

where $e_{i}$ is the number of edges connecting the neighbors of node *i*, reflects the degree to which a node’s immediate neighborhood forms a cohesive subnetwork, often corresponding to functional modules. Complementary to these metrics, Neighborhood Connectivity evaluates the average connectivity of a node’s direct neighbors, providing additional context about its local environment, while Radiality assesses a node’s integration into the overall network architecture.

Conversely, measures such as Eccentricity, $\epsilon_{i}$, defined as the maximum shortest path from node *i* to any other node, underscore the node’s positional centrality by highlighting its remoteness from the network periphery. The Average Shortest Path Length,

(4) $$L_{i}=\frac{1}{n-1}\sum_{j\neq i}d\left(i,j\right),$$

where *n* is the total number of nodes, quantifies the mean distance from node *i* to all others, thereby emphasizing the efficiency of signal transmission. Moreover, Stress, given by

(5) $$S_{i}=\sum_{s\neq i\neq t}\sigma_{st}\left(i\right),$$

captures the cumulative burden placed on a node through its participation in the network’s shortest paths, thereby potentially pinpointing bottlenecks that may compromise network robustness. Finally, the Topological Coefficient, $T_{i}$, gauges the extent to which a node shares common neighbors with other nodes, thus offering insights into its functional redundancy and specialization within the network.

To integrate these diverse topological descriptors into a unified framework, we define the Functional Centrality Index (FCI). The FCI aggregates multiple metrics, each calibrated by a biologically informed weight, into a composite score that reflects the functional significance of a node. Prior to integration, each metric is standardized such that its distribution has a mean of zero and a variance of one, i.e.,

(6) $$z_{m,i}=\frac{m\left(i\right)-\mu_{m}}{\sigma_{m}},$$

where m(i) is the raw value of metric *m* for node *i*, and $\mu_{m}$ and $\sigma_{m}$ are the corresponding mean and standard deviation. For metrics where lower values indicate greater functional importance—such as $L_{i}$, $\epsilon_{i}$, and $S_{i}$—the standardized scores are inverted via multiplication by −1, ensuring that higher *z*-scores uniformly represent superior performance.

The FCI is then computed as the weighted sum of these standardized metrics:

(7) $${\mathrm{FCI}}_{i}=\frac{1}{W}\sum_{m}w_{m}\cdot z_{m,i},$$

with $w_{m}$ denoting the weight attributed to metric *m* and $W=\sum_{m}w_{m}$ serving as the normalization constant. These weights are meticulously chosen to mirror the biological significance of each metric; for instance, Betweenness Centrality may be assigned a relatively high weight to underscore its role in mediating information flow, whereas Closeness Centrality, Clustering Coefficient, Degree, Radiality, and Neighborhood Connectivity capture complementary aspects of both local and global network structure. Inverse metrics such as Eccentricity and Stress, after inversion, contribute to the index in a manner that enhances the interpretability of the overall score.

This formulation of the FCI facilitates the identification of key nodes—whether genes or proteins—that occupy central and functionally critical positions within the network, thereby offering a robust quantitative tool for probing the intricate interplay between network topology and biological function.

#### 3.1.2. Node Clustering

In gene network analysis, the leading eigenvector algorithm is a cornerstone for uncovering community structure through spectral graph theory. Consider a gene interaction network represented by an adjacency matrix A, where each element $A_{ij}$ is defined as 1 if gene *i* interacts with gene *j*, and 0 otherwise. The first step of the algorithm involves constructing the modularity matrix B, with elements given by

(8) $$B_{ij}=A_{ij}-\frac{k_{i}k_{j}}{2m},$$

where $k_{i}=\sum_{j}A_{ij}$ is the degree of node *i* and $m=\frac{1}{2}\sum_{ij}A_{ij}$ represents the total number of edges in the network. This formulation captures the deviation of the observed network from a random configuration that preserves the degree distribution.

A central quantity in this approach is the modularity *Q*, defined as

(9) $$Q=\frac{1}{2m}\;s^{T}B\;s,$$

where the vector s encodes the community assignment of nodes (with $s_{i}=+1$ or −1 indicating membership in one of two communities). Direct maximization of *Q* is a challenging combinatorial problem; however, by relaxing the binary constraint on s, one can approximate the optimal partition via the leading eigenvector of B. Specifically, if v is the eigenvector corresponding to the largest positive eigenvalue $\lambda_{\max}$ of B, then the sign of each component $v_{i}$ guides the community assignment:

(10) $$s_{i}=\left\{\begin{array}{cc} +1, & \mathrm{if}\;v_{i}\geq 0,\\ -1, & \mathrm{if}\;v_{i}<0. \end{array}\right.$$

This spectral method leverages the fact that the leading eigenvector encapsulates the dominant pattern of connectivity, thus revealing the most significant split within the network.

In the context of gene networks—where nodes denote genes and edges represent either physical interactions or functional associations—this algorithm is particularly valuable. It enables the detection of communities that often correspond to functional modules, pathways, or co-regulated gene sets. Such insights are critical for understanding coordinated biological processes and the molecular basis of diseases, such as Parkinson’s disease. The robust mathematical framework provided by the eigen-decomposition of B ensures that the communities identified are statistically significant, effectively distinguishing meaningful structures from random noise, and ultimately offering a quantitative, scalable approach to dissecting the intricate web of gene interactions.

### 3.2. Multi-Modal Graph Neural Network Architecture

We present a deep learning framework that synergistically integrates heterogeneous biological data modalities through a multi-modal graph neural architecture to identify potential therapeutic candidates for Parkinson’s disease. The framework operates on three fundamental biological entities: small molecule drugs represented through chemical descriptors, PPI networks encoding Parkinsonian gene relationships, and functional cluster assignments derived from pathway analysis. Let $D={\left\{d_{i}\right\}}_{i=1}^{N}$ denote the drug set and G=(V,E) represent the PD-specific PPI network with $V={\left\{g_{j}\right\}}_{j=1}^{M}$ genes and edge set E⊆V×V.

#### 3.2.1. Molecular Representation Learning

Each drug molecule $d_{i}$ is encoded through dual complementary representations: a binary Morgan fingerprint $f^{\left(i\right)}\in {\left\{0,1\right\}}^{N_{f}}$ capturing substructure patterns, and a continuous descriptor vector $d^{\left(i\right)}\in R^{N_{d}}$ encoding physicochemical properties. The descriptors undergo standardization ${\hat{d}}^{\left(i\right)}=\left(d^{\left(i\right)}-\mu_{d}\right)⊘\sigma_{d}$ where $\mu_{d}\in R^{N_{d}}$ and $\sigma_{d}\in R^{N_{d}}$ are dataset-wise moment vectors, and ⊘ denotes element-wise division. These features are non-linearly projected into a unified molecular embedding space via

(11) $$h_{mol}^{\left(i\right)}=\Phi \left(\left[\sigma \left(W_{fp}f^{\left(i\right)}+b_{fp}\right)\|\sigma \left(W_{desc}{\hat{d}}^{\left(i\right)}+b_{desc}\right)\right]\right),$$

where $\Phi :R^{N_{f}+N_{d}}\to R^{d_{m}}$ is a multi-layer perceptron (MLP) with batch normalization and dropout, σ denotes the LeakyReLU activation, and ‖ represents feature concatenation. An MLP is a feed-forward neural network built from stacked fully connected (dense) layers separated by nonlinear activation functions; in our framework, compact MLP “heads” transform the fused drug–gene embeddings into final interaction probability scores and uncertainty estimates [50]. LeakyReLU is a variant of the standard Rectified Linear Unit activation that, instead of zeroing out negative inputs, applies a small nonzero slope—e.g., f(x)=max(0.01x,x)—so that negative values still produce a gradient, helping to prevent “dead” neurons during training [51].

#### 3.2.2. Cluster-Aware Gene Network Embedding

The PD-specific PPI network G is enriched with functional cluster information derived from pathway enrichment analysis, partitioning *V* into *K* clusters ${\left\{C_{k}\right\}}_{k=1}^{K}$. Each gene $g_{j}$ receives an initial embedding $e_{j}\in R^{d_{g}}$ from a learnable matrix $E_{gene}\in R^{M\times d_{g}}$, concatenated with its cluster membership vector $c_{j}\in {\left\{0,1\right\}}^{K}$. The cluster-aware graph neural network processes these embeddings through *L* graph attention layers followed by a global transformer layer

(12) $$h_{j}^{\left(l+1\right)}{=∥}_{k=1}^{K_{h}}\sigma \left(\sum_{i\in N(j)}\alpha_{ij}^{\left(k\right)}W^{\left(k\right)}h_{i}^{\left(l\right)}\right)\;\forall l\in \left\{1,\ldots ,L-1\right\},$$

where attention weights $\alpha_{ij}^{\left(k\right)}$ are computed via

(13) $$\alpha_{ij}^{\left(k\right)}={\mathrm{softmax}}_{j}\left(\mathrm{LeakyReLU}\left(a^{(k)⊤}\left[W^{\left(k\right)}h_{i}^{\left(l\right)}\|W^{\left(k\right)}h_{j}^{\left(l\right)}\right]\right)\right).$$

The final transformer layer computes global context-aware embeddings through multi-head attention

(14) $$z_{j}=\Psi \left(\left[\mathrm{MultiHeadAttention}\left(W_{Q}h_{j}^{\left(L\right)},W_{K}H^{\left(L\right)},W_{V}H^{\left(L\right)}\right)\|c_{j}\right]\right),$$

where $H^{\left(L\right)}\in R^{M\times d_{h}}$ is the node embedding matrix and Ψ is a projection network.

#### 3.2.3. Uncertainty-Aware Drug-Gene Interaction Modeling

The interaction likelihood between drug $d_{i}$ and gene $g_{j}$ is modeled through a dual cross-attention mechanism

(15) $$A_{i,j}=\frac{\left(W_{Q}h_{mol}^{\left(i\right)}\right){\left(W_{K}z_{j}\right)}^{⊤}}{\sqrt{d_{k}}}\;a_{i,j}=\mathrm{softmax}\left(A_{i,j}\right)$$

yielding attended representations $h_{d\to g}=a_{i,j}W_{V}z_{j}$ and $h_{g\to d}=a_{j,i}W_{V}h_{mol}^{\left(i\right)}$. The interaction score $s_{i,j}\in \left[0,1\right]$ and aleatoric uncertainty $u_{i,j}\in R^{+}$ are computed as

(16) $$s_{i,j}=\Omega \left(\left[h_{d\to g}\|h_{g\to d}\right]\right),\;u_{i,j}=\mathrm{softplus}\left(\Gamma \left(\left[h_{d\to g}\|h_{g\to d}\right]\right)\right),$$

where Ω and Γ are MLPs with single-dimensional outputs.

#### 3.2.4. Multi-Objective Optimization

The model simultaneously optimizes three objectives

1. Drug-Target Interaction Prediction:
  (17) $$L_{DTI}=\sum_{\left(i,j\right)}\frac{\mathrm{BCE}(s_{i,j},y_{i,j})}{u_{i,j}}+\lambda_{u}\sum_{\left(i,j\right)}\log u_{i,j}$$
2. PD Gene Classification:
  (18) $$L_{gene}=\lambda_{g}\sum_{j}\mathrm{BCE}\left(\sigma \left(\Theta \left(z_{j}\right)\right),y_{j}\right)$$
3. Molecular Autoencoding:
  (19) $$L_{recon}=\lambda_{r}\left(∥\Upsilon_{fp}\left(h_{mol}^{\left(i\right)}\right)-f^{\left(i\right)}{∥}_{1}+{∥\Upsilon_{desc}\left(h_{mol}^{\left(i\right)}\right)-{\hat{d}}^{\left(i\right)}∥}_{2}^{2}\right)$$
  where BCE denotes Binary Cross-Entropy.

The composite loss $L_{total}=L_{DTI}+L_{gene}+L_{recon}$ is minimized through stochastic gradient descent with adaptive uncertainty weighting.

#### 3.2.5. Uncertainty-Quantified Drug Prioritization

For candidate screening, we compute epistemic uncertainty via Monte Carlo dropout

(20) $${\bar{s}}_{i,j}=\frac{1}{T}\sum_{t=1}^{T}s_{i,j}^{\left(t\right)},\;\sigma_{i,j}^{2}=\frac{1}{T}\sum_{t=1}^{T}{\left(s_{i,j}^{\left(t\right)}-{\bar{s}}_{i,j}\right)}^{2},.$$

Drugs are ranked by aggregating scores across PD target genes $G_{target}$

(21) $$\mathrm{Rank}\left(d_{i}\right)=\underset{\mathrm{Interaction}\;\mathrm{Affinity}}{\underset{︸}{\frac{1}{|G_{target}|}\sum_{g_{j}\in G_{target}}{\bar{s}}_{i,j}}}-\lambda_{r}\underset{\mathrm{Total}\;\mathrm{Uncertainty}}{\underset{︸}{\frac{1}{|G_{target}|}\sum_{g_{j}\in G_{target}}\sqrt{u_{i,j}^{2}+\sigma_{i,j}^{2}}}}.$$

This dual-component ranking metric balances interaction likelihood against prediction confidence, enabling prioritization of high-affinity candidates with robust evidence. The framework’s multi-modal integration of chemical, genomic, and pathway data through deep geometric learning provides a systematic approach for identifying Parkinson’s therapeutics with quantified uncertainty estimates.

**Figure 10:**
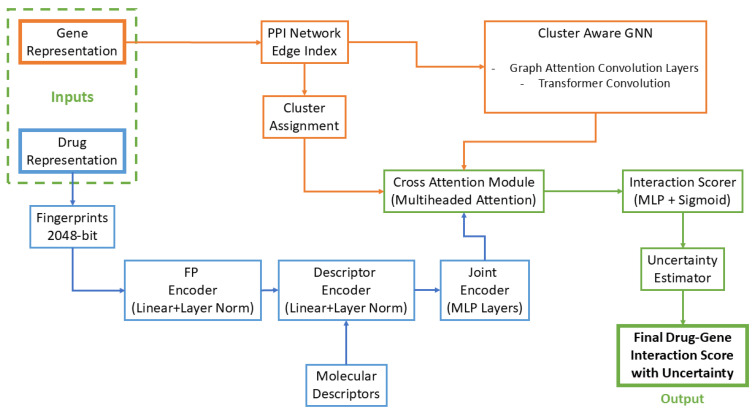
Multi-Modal graph neural network framework.

The proposed Multi-Modal Graph Neural Network architecture integrates diverse molecular and genetic representations to effectively model the complex interactions between candidate drugs and Parkinson’s disease target genes. This integration addresses a fundamental challenge in computational drug discovery: the synthesis of heterogeneous data types within a unified predictive framework. Figure 10 present the complete framework we present in this study.

In our multi-modal GNN pipeline, each small molecule is first featurized by computing a 2048-bit Morgan fingerprint alongside a panel of 1826 physicochemical descriptors; these two vectors are jointly embedded by the MolecularEncoder into a 512-dimensional drug representation. In parallel, each candidate gene is mapped to a learned embedding and then refined by passing through the ClusterAwareGNN, which propagates information across the PD-specific PPI network while leveraging module (cluster) assignments to bias the message-passing dynamics. The resulting drug and gene embeddings enter a Cross-Attention module, which aligns and fuses their features into a combined vector. Finally, a lightweight MLP head converts this fused embedding into (i) a sigmoid-activated interaction probability and (ii) an uncertainty estimate, thereby producing both a confidence-weighted prediction and an accompanying measure of model certainty.

The Multi-Modal Graph Neural Network was implemented with precise architectural specifications to optimize performance for Parkinson’s disease drug repurposing. The molecular encoder processes 2048-bit Morgan fingerprints (radius = 2) and 200-dimensional molecular descriptors through parallel pathways with hidden dimensions of 512 units. Each encoder employs a two-layer architecture with LeakyReLU activation (negative slope = 0.2) and dropout regularization (p=0.2), followed by LayerNorm—a normalization method that standardizes the summed inputs to each neuron across its features rather than across the batch, ensuring consistent scaling even when batch sizes vary. Input fingerprints undergo binarization prior to processing, while descriptors are scaled with scikit-learn’s StandardScaler—a preprocessing tool that applies z-score normalization by subtracting each feature’s mean and dividing by its standard deviation to yield zero-mean, unit-variance data—before being fed into the network.

The gene representation module initializes embeddings for all genes using Xavier uniform initialization to mitigate training instability. These embeddings are processed through a dual-layer neural network with LayerNorm and LeakyReLU activations, maintaining consistent 512-dimensional representations. For network integration, we employ a three-layer graph architecture comprising two Graph Attention Convolution GATConv, a graph-neural-network layer that uses attention mechanisms on graph edges, layers (8 attention heads each, with concatenation aggregation) followed by a final TransformerConv, a graph-convolution layer inspired by the Transformer architecture, layer for global context. The PPI network contains 83,426 validated edges after filtering, which are processed as an undirected graph with self-loops removed to prevent feature oversmoothing.

Our cluster-aware projection incorporates 5-dimensional one-hot encoded cluster assignments derived from network modularity analysis. This information is concatenated with node features and transformed through a linear layer (517→512 dimensions) with LayerNorm and dropout (p=0.2). During subgraph processing, we implement edge index batching with proper node relabeling to ensure computational efficiency, achieving processing rates of approximately 5500 drug-gene pairs per second on an NVIDIA ADA 3000 RTX, a high-performance graphics processing unit (GPU) from NVIDIA’s Ada Loveless architecture line.

The cross-attention mechanism implements an 8-head attention structure (head dimension = 64) with scaled dot-product attention (scaling factor = 1/8) and dropout (p=0.1) applied to attention weights. Query, key, and value projections maintain consistent dimensions (512→512) through independent linear transformations. The interaction scorer comprises a three-layer network (1024→512→256→1) with LeakyReLU activations, LayerNorm, and dropout (p=0.2), producing unbounded scores that undergo sigmoid activation for final [0,1] bounded outputs.

For uncertainty quantification, we implement a parallel head with identical architecture to the scorer but terminating with a sigmoid activation directly. During inference, we maintain dropout in evaluation mode and perform 10 stochastic forward passes, calculating the predictive mean and variance to derive aleatoric uncertainty estimates. Uncertainty scores are scaled by a factor of 0.5 to constrain the upper bound to 0.5, corresponding to maximum prediction uncertainty.

The model was trained using Adam optimization ($\beta_{1}=0.9$, $\beta_{2}=0.999$) with a learning rate of $10^{-4}$ and batch size of 64. For regularization, we employ $L_{2}$ weight decay ($\Lambda =10^{-5}$) and early stopping (patience = 10 epochs, monitoring the area under the receiver operating characteristic curve (AUROC) on the validation set). The loss function combines binary cross-entropy with uncertainty-weighted regularization ($\lambda_{uncertainty}=0.1$) to penalize overconfident predictions. Self-supervised pretraining was conducted on 9816 DrugBank compounds with a reconstruction loss combining BCE for fingerprints and mean squared error (MSE) for descriptors, with a pretraining weight of $\lambda_{pretrain}=0.3$ relative to the main task loss.

During drug screening, we segment the database into batches of 64 compounds with GPU memory management to handle the complete DrugBank library of 11,292 compounds. Z-score normalization is applied post-hoc to raw interaction scores, and compounds are filtered by uncertainty threshold (τ=0.5, relaxed to τ=0.7 if fewer than 10 compounds pass the initial filter). The final ranking incorporates both normalized scores and uncertainty metrics, with empirical percentile calculations based on rank position rather than theoretical normal distribution assumptions.

## 4. Conclusions

This study presents an integrative computational framework that combines network biology with a multi-modal graph neural network to identify and prioritize multi-target drug candidates for Parkinson’s disease. By harnessing protein–protein interaction networks, advanced clustering methods, and gene set enrichment analyses, our approach elucidates key molecular modules driving PD pathogenesis. The incorporation of polypharmacology through GNN predictions—augmented by uncertainty quantification—has enabled the identification of several promising repurposing candidates that simultaneously target critical nodes across diverse pathways, including lysosomal function, mitochondrial integrity, and neuroinflammation.

The findings reinforce the concept that Parkinson’s disease is a multifactorial network disorder, where effective therapeutic interventions must address interconnected biological processes rather than single targets. Although some predicted compounds, such as vitamin E derivatives and clinically established agents, offer encouraging translational potential, others highlight the need for further medicinal chemistry optimization to overcome safety or pharmacokinetic limitations.

Future work should focus on validating these computational predictions through rigorous in vitro and in vivo assays, refining drug formulations to improve central nervous system delivery, and ultimately advancing the most promising candidates into clinical evaluation. Overall, our study provides a novel, systems-level blueprint for drug repurposing in complex neurodegenerative diseases, offering new avenues for the development of multi-target therapies in Parkinson’s disease.

## Figures and Tables

**Table 1:** Top terms of GO enrichment results for Cluster 1.

GO ID	GO Term	*p*-Value	FDR	Cluster Frequency (%)	Background Frequency (%)	Enrichment Factor
50220	prostaglandin-E synthase activity	$1.65\times 10^{-5}$	$9.76\times 10^{-3}$	0.17%	10.0%	0.02
8440	inositol trisphosphate 3-kinase activity	$5.74\times 10^{-5}$	$9.76\times 10^{-3}$	0.17%	10.0%	0.02
44255	cellular lipid metabolic process	$2.88\times 10^{-5}$	$9.76\times 10^{-3}$	0.17%	10.0%	0.02
6629	lipid metabolic process	$5.30\times 10^{-5}$	$9.76\times 10^{-3}$	0.17%	10.0%	0.02
51766	inositol trisphosphate kinase activity	$7.65\times 10^{-5}$	0.0104	0.17%	10.0%	0.02
30384	phosphoinositide metabolic process	$3.02\times 10^{-4}$	0.0236	0.17%	10.0%	0.02
6633	fatty acid biosynthetic process	$4.17\times 10^{-4}$	0.0236	0.17%	10.0%	0.02
46457	prostanoid biosynthetic process	$2.47\times 10^{-4}$	0.0236	0.17%	10.0%	0.02
16044	cellular membrane organization	$4.07\times 10^{-4}$	0.0236	0.17%	10.0%	0.02
61024	membrane organization	$4.12\times 10^{-4}$	0.0236	0.17%	10.0%	0.02
1516	prostaglandin biosynthetic process	$2.47\times 10^{-4}$	0.0236	0.17%	10.0%	0.02
15035	protein disulfide oxidoreductase activity	$3.25\times 10^{-4}$	0.0236	0.17%	10.0%	0.02
15036	disulfide oxidoreductase activity	$5.66\times 10^{-4}$	0.0264	0.17%	10.0%	0.02
46578	regulation of Ras protein signal transduction	$6.11\times 10^{-4}$	0.0264	0.17%	10.0%	0.02
6692	prostanoid metabolic process	$6.22\times 10^{-4}$	0.0264	0.17%	10.0%	0.02
6693	prostaglandin metabolic process	$6.22\times 10^{-4}$	0.0264	0.17%	10.0%	0.02
917	barrier septum formation	$1.69\times 10^{-3}$	0.0395	0.17%	10.0%	0.02
47323	[3-methyl-2-oxobutanoate dehydrogenase (acetyl-transferring)] kinase activity	$1.69\times 10^{-3}$	0.0395	0.17%	10.0%	0.02
4316	3-oxoacyl-[acyl-carrier-protein] reductase activity	$1.69\times 10^{-3}$	0.0395	0.17%	10.0%	0.02
19171	3-hydroxyacyl-[acyl-carrier-protein] dehydratase activity	$1.69\times 10^{-3}$	0.0395	0.17%	10.0%	0.02

**Table 2:** Top terms of GO enrichment results for Cluster 2.

GO ID	GO Term	*p*-Value	FDR	Cluster Frequency (%)	Background Frequency (%)	Enrichment Factor
48489	synaptic vesicle transport	$2.08\times 10^{-5}$	0.0149	0.17%	10.0%	0.02
43005	neuron projection	$3.17\times 10^{-5}$	0.0149	0.17%	10.0%	0.02
17157	regulation of exocytosis	$5.47\times 10^{-5}$	0.0172	0.17%	10.0%	0.02
44456	synapse part	$8.25\times 10^{-5}$	0.0185	0.17%	10.0%	0.02
1963	synaptic transmission, dopaminergic	$9.83\times 10^{-5}$	0.0185	0.17%	10.0%	0.02
60341	regulation of cellular localization	$1.44\times 10^{-4}$	0.0202	0.17%	10.0%	0.02
4385	guanylate kinase activity	$1.50\times 10^{-4}$	0.0202	0.17%	10.0%	0.02
5886	plasma membrane	$3.96\times 10^{-4}$	0.0249	0.17%	10.0%	0.02
80010	regulation of oxygen and reactive oxygen species metabolic process	$3.25\times 10^{-4}$	0.0249	0.17%	10.0%	0.02
6836	neurotransmitter transport	$3.63\times 10^{-4}$	0.0249	0.17%	10.0%	0.02
8021	synaptic vesicle	$3.76\times 10^{-4}$	0.0249	0.17%	10.0%	0.02
44463	cell projection part	$2.80\times 10^{-4}$	0.0249	0.17%	10.0%	0.02
19226	transmission of nerve impulse	$2.45\times 10^{-4}$	0.0249	0.17%	10.0%	0.02
45202	synapse	$3.39\times 10^{-4}$	0.0249	0.17%	10.0%	0.02
30424	axon	$2.17\times 10^{-4}$	0.0249	0.17%	10.0%	0.02
23046	signaling process	$4.91\times 10^{-4}$	0.0254	0.17%	10.0%	0.02
42417	dopamine metabolic process	$5.13\times 10^{-4}$	0.0254	0.17%	10.0%	0.02
23060	signal transmission	$4.91\times 10^{-4}$	0.0254	0.17%	10.0%	0.02
46928	regulation of neurotransmitter secretion	$5.13\times 10^{-4}$	0.0254	0.17%	10.0%	0.02
6810	transport	$5.64\times 10^{-4}$	0.0266	0.17%	10.0%	0.02

**Table 3:** Top terms of GO enrichment results for Cluster 3.

GO ID	GO Term	*p*-Value	FDR	Cluster Frequency (%)	Background Frequency (%)	Enrichment Factor
31077	post-embryonic camera-type eye development	$1.76\times 10^{-5}$	$5.45\times 10^{-3}$	0.17%	10.0%	0.02
33153	T cell receptor V(D)J recombination	$1.76\times 10^{-5}$	$5.45\times 10^{-3}$	0.17%	10.0%	0.02
2568	somatic diversification of T cell receptor genes	$1.76\times 10^{-5}$	$5.45\times 10^{-3}$	0.17%	10.0%	0.02
2681	somatic recombination of T cell receptor gene segments	$1.76\times 10^{-5}$	$5.45\times 10^{-3}$	0.17%	10.0%	0.02
30030	cell projection organization	$3.36\times 10^{-5}$	$8.34\times 10^{-3}$	0.17%	10.0%	0.02
323	lytic vacuole	$5.05\times 10^{-5}$	$8.94\times 10^{-3}$	0.17%	10.0%	0.02
5764	lysosome	$5.05\times 10^{-5}$	$8.94\times 10^{-3}$	0.17%	10.0%	0.02
32501	multicellular organismal process	$7.17\times 10^{-5}$	0.0111	0.17%	10.0%	0.02
48569	post-embryonic organ development	$1.05\times 10^{-4}$	0.0130	0.17%	10.0%	0.02
5773	vacuole	$1.01\times 10^{-4}$	0.0130	0.17%	10.0%	0.02
51000	positive regulation of nitric-oxide synthase activity	$1.31\times 10^{-4}$	0.0137	0.17%	10.0%	0.02
48513	organ development	$1.32\times 10^{-4}$	0.0137	0.17%	10.0%	0.02
2376	immune system process	$1.67\times 10^{-4}$	0.0138	0.17%	10.0%	0.02
33151	V(D)J recombination	$1.60\times 10^{-4}$	0.0138	0.17%	10.0%	0.02
5886	plasma membrane	$1.58\times 10^{-4}$	0.0138	0.17%	10.0%	0.02
31982	vesicle	$1.84\times 10^{-4}$	0.0143	0.17%	10.0%	0.02
7275	multicellular organismal development	$1.98\times 10^{-4}$	0.0144	0.17%	10.0%	0.02
51496	positive regulation of stress fiber assembly	$2.64\times 10^{-4}$	0.0182	0.17%	10.0%	0.02
32770	positive regulation of monooxygenase activity	$3.04\times 10^{-4}$	0.0192	0.17%	10.0%	0.02
45471	response to ethanol	$3.09\times 10^{-4}$	0.0192	0.17%	10.0%	0.02

**Table 4:** Top terms of GO enrichment results for Cluster 4.

GO ID	GO Term	*p*-Value	FDR	Cluster Frequency (%)	Background Frequency (%)	Enrichment Factor
8139	nuclear localization sequence binding	$4.75\times 10^{-5}$	0.0236	0.12%	10.0%	0.01
6607	NLS-bearing substrate import into nucleus	$1.03\times 10^{-4}$	0.0255	0.12%	10.0%	0.01
17048	Rho GTPase binding	$5.29\times 10^{-4}$	0.0352	0.12%	10.0%	0.01
18024	histone-lysine N-methyltransferase activity	$5.66\times 10^{-4}$	0.0352	0.12%	10.0%	0.01
16279	protein-lysine N-methyltransferase activity	$5.66\times 10^{-4}$	0.0352	0.12%	10.0%	0.01
5048	signal sequence binding	$3.02\times 10^{-4}$	0.0352	0.12%	10.0%	0.01
5654	nucleoplasm	$5.53\times 10^{-4}$	0.0352	0.12%	10.0%	0.01
16278	lysine N-methyltransferase activity	$5.66\times 10^{-4}$	0.0352	0.12%	10.0%	0.01
31252	cell leading edge	$9.05\times 10^{-4}$	0.0450	0.12%	10.0%	0.01
42054	histone methyltransferase activity	$8.62\times 10^{-4}$	0.0450	0.12%	10.0%	0.01

**Table 5:** Top terms of GO enrichment results for Cluster 5.

GO ID	GO Term	*p*-Value	FDR	Cluster Frequency (%)	Background Frequency (%)	Enrichment Factor
4631	phosphomevalonate kinase activity	$5.06\times 10^{-4}$	0.0272	0.05%	10.0%	0.01
5739	mitochondrion	$1.83\times 10^{-4}$	0.0272	0.05%	10.0%	0.01
6768	biotin metabolic process	$5.06\times 10^{-4}$	0.0272	0.05%	10.0%	0.01
5737	cytoplasm	$5.01\times 10^{-4}$	0.0272	0.05%	10.0%	0.01
4485	methylcrotonoyl-CoA carboxylase activity	$1.01\times 10^{-3}$	0.0435	0.05%	10.0%	0.01

**Table 6:** Drugs targeting multiple Parkinson’s genes.

Drug	DrugBank ID	Targets	z-Score	Percentile (%)	Probability (%)	Uncertainty
Dithiazanine	DB11516	GAK, KANSL1	3.01	99.9	94.7	0.1425
Ceftolozane	DB09050	TMEM175, RIT2	2.96	99.9	89.1	0.2589
DL-alpha-Tocopherol	DB14476	BCKDK, MAPT	3.15	100.0	93.6	0.2629
Bromisoval	DB13370	LRRK2, APOE	3.37	100.0	98.6	0.2198
Imidurea	DB14075	APOE, CRLS1	2.97	99.9	94.8	0.2147
Medronic acid	DB14078	GPNMB, BST1	2.97	99.9	85.8	0.2464
Modufolin	DB12676	NUCKS1, WWOX	2.83	99.8	89.6	0.3004

## Data Availability

The original contributions presented in the study are included in the article, further inquiries can be directed to the corresponding author.

## Appendix A

**Table A1:** Top 10 drugs predicted by Multi-Modal GNN for five genes with highest FCI scores in Cluster 1.

Drug Name	DrugBank ID	Raw Scpre	z-Score	Uncertainty	Percentile	Elite Percentile	Probability	Gene
Glycochenodeoxycholic Acid	DB02123	0.8317	3.676	0.2944	100.0	100.0	100.0	DGKQ
Vilazodone	DB06684	0.7994	3.2732	0.3351	99.9919	99.8374	94.5108	DGKQ
Ferrous chloride	DB13569	0.7989	3.268	0.343	99.9837	99.6748	94.4401	DGKQ
S-Butyryl-Cystein	DB02160	0.7959	3.2302	0.3005	99.9756	99.5122	93.926	DGKQ
2-{4-[4-(4-Chloro-Phenoxy)-Benzenesulfonyl] -Tetrahydro-Pyran-4-Yl}-N-Hydroxy-Acetamide	DB02049	0.7891	3.1451	0.3196	99.9675	99.3496	92.7659	DGKQ
Flutemetamol	DB15058	0.7808	3.0415	0.2691	99.9593	99.187	91.3541	DGKQ
Netarsudil	DB13931	0.7769	2.9937	0.2364	99.9512	99.0244	90.7025	DGKQ
D-Borneol	DB17066	0.7753	2.9737	0.2433	99.9431	98.8618	90.4295	DGKQ
Ticagrelor	DB08816	0.7723	2.9359	0.2601	99.9349	98.6992	89.9147	DGKQ
6-Chloro-2-(2-Hydroxy-Biphenyl-3-Yl) -1h-Indole-5-Carboxamidine	DB03865	0.7714	2.9256	0.2983	99.9268	98.5366	89.774	DGKQ
Indobufen	DB12545	0.8207	3.5023	0.3538	100.0	100.0	100.0	GAK
Netarsudil	DB13931	0.8005	3.2541	0.3399	99.9919	99.8374	96.4035	GAK
alpha-D-Xylopyranose	DB03389	0.7999	3.2471	0.3363	99.9837	99.6748	96.3025	GAK
Mibefradil	DB01388	0.7891	3.1136	0.249	99.9756	99.5122	94.3682	GAK
Fluprednisolone	DB09378	0.7826	3.0336	0.3111	99.9675	99.3496	93.2086	GAK
Ciclesonide	DB01410	0.7801	3.0036	0.3088	99.9593	99.187	92.7734	GAK
Imiglitazar	DB12511	0.7801	3.0027	0.3701	99.9512	99.0244	92.7611	GAK
L-Baclofen	DB12098	0.7747	2.9364	0.3532	99.9431	98.8618	91.8	GAK
3-(4-nitrophenyl)-1H-pyrazole	DB08695	0.7736	2.9233	0.2412	99.9349	98.6992	91.6099	GAK
Flortaucipir	DB15033	0.7723	2.908	0.3114	99.9268	98.5366	91.3885	GAK
Cortisone acetate	DB01380	0.8146	3.2992	0.3751	100.0	100.0	100.0	GBA
Perillyl alcohol	DB15289	0.8067	3.202	0.386	99.9919	99.8374	98.6197	GBA
2-(2-{2-[(BIPHENYL-4-YLMETHYL)-AMINO] -3-MERCAPTO-PENTANOYLAMINO}-ACETYLAMINO) -3-METHYL-BUTYRIC ACID METHYL ESTER	DB08643	0.8015	3.1366	0.3976	99.9837	99.6748	97.6906	GBA
alpha-N-Acetyl-D-galactosamine	DB03567	0.7976	3.0891	0.3389	99.9756	99.5122	97.0158	GBA
Landiolol	DB12212	0.7936	3.0387	0.3038	99.9675	99.3496	96.2997	GBA
(1S)-2-[(2S,5R)-2-(AMINOMETHYL) -5-ETHYNYLPYRROLIDIN-1-YL]-1 -CYCLOPENTYL-2-OXOETHANAMINE	DB07356	0.7927	3.0278	0.3095	99.9593	99.187	96.1442	GBA
N-(2-(((5-CHLORO-2-PYRIDINYL)AMINO)SULFONYL) PHENYL)-4-(2-OXO-1(2H)-PYRIDINYL)BENZAMIDE	DB07800	0.7918	3.0163	0.3405	99.9512	99.0244	95.9808	GBA
LY-294002	DB02656	0.7884	2.9744	0.3492	99.9431	98.8618	95.3856	GBA
N-{2-[4-(AMINOSULFONYL)PHENYL]ETHYL}ACETAMIDE	DB08155	0.7848	2.9302	0.2484	99.9349	98.6992	94.7587	GBA
Quinolinic Acid	DB01796	0.7848	2.9302	0.3113	99.9268	98.5366	94.7577	GBA
(1R,2R,3S,4R,6S)-3,4,6-Trihydroxy-5 -{[(S)-hydroxy(3-hydroxy-2-oxopropoxy)phosphoryl]oxy} -1,2-cyclohexanediyl bis[dihydrogen (phosphate)]	DB02028	0.8299	3.7466	0.2958	100.0	100.0	100.0	TMEM175
CTS-1027	DB08490	0.8296	3.7434	0.2995	99.9919	99.8374	99.9583	TMEM175
Vibegron	DB14895	0.8257	3.6942	0.3392	99.9837	99.6748	99.3229	TMEM175
Ranolazine	DB00243	0.8225	3.6551	0.3184	99.9756	99.5122	98.8164	TMEM175
3-nitro-L-tyrosine	DB03867	0.8093	3.4922	0.3465	99.9675	99.3496	96.7083	TMEM175
3-hydroxyglutaric acid	DB04594	0.8037	3.4226	0.2793	99.9593	99.187	95.8081	TMEM175
LG-100268	DB01941	0.7891	3.2414	0.2456	99.9512	99.0244	93.4636	TMEM175
Atecegatran metoxil	DB12507	0.782	3.1547	0.3315	99.9431	98.8618	92.3408	TMEM175
(1S)-1-AMINO-2-(1H-INDOL-3-YL)ETHANOL	DB08649	0.7792	3.1189	0.3247	99.9349	98.6992	91.8777	TMEM175
Topiramate	DB00273	0.777	3.0928	0.2848	99.9268	98.5366	91.5404	TMEM175
Ramelteon	DB00980	0.8421	3.7712	0.3392	100.0	100.0	100.0	BCKDK
Indomethacin	DB00328	0.7894	3.119	0.3447	99.9919	99.8374	91.6803	BCKDK
(1S,2S,5S)2-(4-GLUTARIDYLBENZYL) -5-PHENYL-1-CYCLOHEXANOL	DB07909	0.7893	3.1178	0.3616	99.9837	99.6748	91.6656	BCKDK
Zinc Substituted Heme C	DB04249	0.7863	3.081	0.3536	99.9756	99.5122	91.1965	BCKDK
3-{[(3-FLUORO-3’-METHOXYBIPHENYL-4-YL)AMINO] CARBONYL}THIOPHENE-2-CARBOXYLIC ACID	DB07976	0.7831	3.0412	0.3324	99.9675	99.3496	90.6886	BCKDK
Pipequaline	DB13991	0.7806	3.0107	0.2865	99.9593	99.187	90.2999	BCKDK
(1S,7S,8S,8AR)-1,2,3,7,8,8A-HEXAHYDRO-7-METHYL -8-[2-[(2R,4R)-TETRAHYDRO-4-HY DROXY-6-OXO-2H-PYRAN -2-YL]ETHYL]-1-NAPHTHALENOL	DB08224	0.7727	2.913	0.365	99.9512	99.0244	89.0531	BCKDK
Fluocortolone	DB08971	0.7698	2.8768	0.3908	99.9431	98.8618	88.5921	BCKDK
Ganaplacide	DB16173	0.7693	2.8711	0.3198	99.9349	98.6992	88.5191	BCKDK
Anisotropine methylbromide	DB00517	0.7655	2.8238	0.3696	99.9268	98.5366	87.9157	BCKDK

**Table A2:** Top 10 drugs predicted by Multi-Modal GNN for five genes with highest FCI scores in Cluster 2.

Drug Name	DrugBank ID	Raw Score	z-Score	Uncertainty	Percentile	Elite Percentile	Probability	Gene
Clorotepine	DB15971	0.8113	3.4489	0.3352	100.0	100.0	100.0	MAPT
Oxtriphylline	DB01303	0.7911	3.2018	0.2308	99.9919	99.8374	96.3726	MAPT
Verdoheme	DB04803	0.786	3.1402	0.3065	99.9837	99.6748	95.468	MAPT
Dodecyltrimethylammonium	DB02779	0.7725	2.9742	0.2759	99.9756	99.5122	93.0316	MAPT
R-95845	DB07327	0.7703	2.9473	0.3677	99.9675	99.3496	92.6366	MAPT
TP-1287	DB18104	0.7685	2.9257	0.271	99.9593	99.187	92.3184	MAPT
TG100-115	DB05552	0.7669	2.9061	0.3139	99.9512	99.0244	92.0308	MAPT
Bamethan	DB13206	0.7627	2.8551	0.3252	99.9431	98.8618	91.2826	MAPT
4-{5-[(1Z)-1-(2-IMINO-4-OXO-1,3 -THIAZOLIDIN-5-YLIDENE)ETHYL] -2-FURYL}BENZENESULFONAMIDE	DB07540	0.7618	2.8442	0.319	99.9349	98.6992	91.1232	MAPT
Phenaglycodol	DB19373	0.7605	2.8277	0.3134	99.9268	98.5366	90.8806	MAPT
Desmopressin	DB00035	0.7938	3.3745	0.3725	100.0	100.0	100.0	SNCA
Simenepag isopropyl	DB12977	0.7846	3.2621	0.2972	99.9919	99.8374	98.3222	SNCA
Bolasterone	DB01471	0.7774	3.1734	0.3701	99.9837	99.6748	96.9989	SNCA
Sodium tetradecyl sulfate	DB00464	0.7735	3.1264	0.24	99.9756	99.5122	96.2981	SNCA
Florbenazine F-18	DB14945	0.7687	3.0674	0.2229	99.9675	99.3496	95.417	SNCA
MALP-2	DB18482	0.768	3.059	0.2854	99.9593	99.187	95.292	SNCA
Metoprine	DB04655	0.7666	3.042	0.3034	99.9512	99.0244	95.0387	SNCA
Medifoxamine	DB13219	0.7645	3.0162	0.3691	99.9431	98.8618	94.6535	SNCA
Nimesulide	DB04743	0.7631	2.9993	0.3322	99.9349	98.6992	94.4003	SNCA
Chlorotoxin I-131	DB05462	0.7629	2.997	0.3783	99.9268	98.5366	94.3666	SNCA
(R)-methylmalonyl-CoA	DB04045	0.789	2.9323	0.3501	100.0	100.0	100.0	RIT2
Gestrinone	DB11619	0.7884	2.9254	0.3161	99.9919	99.8374	99.8871	RIT2
2-(2,4-DICHLOROPHENOXY) -5-(PYRIDIN-2-YLMETHYL)PHENOL	DB07287	0.7807	2.8289	0.3101	99.9837	99.6748	98.3112	RIT2
Tetrazepam	DB13324	0.7784	2.7995	0.2862	99.9756	99.5122	97.8325	RIT2
Tetragalacturonic acid hydroxymethylester	DB13621	0.7765	2.7756	0.2442	99.9675	99.3496	97.4415	RIT2
Nojirimycine Tetrazole	DB02471	0.7761	2.771	0.3202	99.9593	99.187	97.3674	RIT2
Deudextromethorphan	DB19054	0.7737	2.7408	0.3655	99.9512	99.0244	96.8746	RIT2
Deoxyamidinoproclavaminic acid	DB02475	0.7719	2.7174	0.2686	99.9431	98.8618	96.492	RIT2
Levobetaxolol	DB09351	0.7715	2.7127	0.3006	99.9349	98.6992	96.4154	RIT2
Prasterone enantate	DB16625	0.7712	2.7092	0.3265	99.9268	98.5366	96.3585	RIT2
2c-Methyl-D-Erythritol 2,4 -Cyclodiphosphate	DB03961	0.8126	3.3758	0.3139	100.0	100.0	100.0	PRKN
Sanfetrinem cilexetil	DB19135	0.8105	3.3497	0.3219	99.9919	99.8374	99.6261	PRKN
Acetarsol	DB13268	0.808	3.3186	0.2309	99.9837	99.6748	99.1797	PRKN
N-(4-{[(3S)-3-(dimethylamino)pyrrolidin-1-yl] carbonyl}phenyl)-5-fluoro-4-[2-methyl-1 -(1-methylethyl)-1H-imidazol-5-yl]pyrimidin -2-amine	DB07936	0.7921	3.1223	0.3362	99.9756	99.5122	96.3617	PRKN
(1R,4S,7AS)-1-(1-FORMYLPROP-1-EN-1-YL) -4-METHOXY-2,4,5,6,7,7A-HEXAHYDRO -1H-ISOINDOLE-3-CARBOXYLIC ACID	DB08110	0.7845	3.0285	0.2737	99.9675	99.3496	95.0164	PRKN
Halothane	DB01159	0.7844	3.0265	0.2784	99.9593	99.187	94.987	PRKN
Phendimetrazine	DB01579	0.7841	3.0227	0.301	99.9512	99.0244	94.9334	PRKN
Uridine diphosphate glucose	DB01861	0.7838	3.0192	0.3706	99.9431	98.8618	94.8823	PRKN
Icaritin	DB12672	0.7794	2.9643	0.3944	99.9349	98.6992	94.0949	PRKN
Indralin	DB18220	0.7774	2.9395	0.354	99.9268	98.5366	93.7393	PRKN
Genz-10850	DB04289	0.802	3.194	0.3738	100.0	100.0	100.0	STX1B
Brasofensine	DB04857	0.7981	3.1468	0.3115	99.9919	99.8374	99.2901	STX1B
5-(3-{3-[3-HYDROXY-2-(METHOXYCARBONYL) PHENOXY]PROPENYL}PHENYL) -4-(HYDROXYMETHYL)ISOXAZOLE -3-CARBOXYLIC ACID	DB08001	0.7884	3.0266	0.3049	99.9837	99.6748	97.4823	STX1B
Tavapadon	DB14899	0.7817	2.9442	0.2889	99.9756	99.5122	96.2434	STX1B
3-[(9H-fluoren-9-ylideneamino)oxy]propanoic acid	DB07240	0.78	2.9226	0.2985	99.9675	99.3496	95.9172	STX1B
Pirepemat	DB19165	0.7799	2.9212	0.3235	99.9593	99.187	95.8969	STX1B
Epibatidine	DB07720	0.7785	2.9049	0.37	99.9512	99.0244	95.651	STX1B
Clonazepam	DB01068	0.7766	2.8814	0.2408	99.9431	98.8618	95.2977	STX1B
Osalmid	DB16273	0.773	2.8367	0.3213	99.9349	98.6992	94.626	STX1B
4,5-Dimethyl-1,2-phenylenediamine	DB03180	0.7725	2.8304	0.3054	99.9268	98.5366	94.5305	STX1B

**Table A3:** Top 10 drugs predicted by Multi-Modal GNN for five genes with highest FCI scores in Cluster 3.

Drug Name	DrugBank ID	Raw Score	z-Score	Uncertainty	Percentile	Elite Percentile	Probability	Gene
Henatinib	DB13019	0.8187	3.1505	0.3136	100.0	100.0	100.0	LRRK2
Betameprodine	DB01552	0.8183	3.146	0.3601	99.9919	99.8374	99.9323	LRRK2
Vatalanib	DB04879	0.8087	3.0278	0.3496	99.9837	99.6748	98.158	LRRK2
Histapyrrodine	DB13479	0.8045	2.9753	0.2981	99.9756	99.5122	97.3698	LRRK2
P-Anisic Acid	DB02795	0.7954	2.8626	0.3591	99.9675	99.3496	95.6782	LRRK2
Limonene, (+/-)-	DB19146	0.7935	2.8393	0.2723	99.9593	99.187	95.3283	LRRK2
Cemdomespib	DB18953	0.793	2.8332	0.2955	99.9512	99.0244	95.2376	LRRK2
(3S)-1-CYCLOHEXYL-5-OXO -N-PHENYLPYRROLIDINE -3-CARBOXAMIDE	DB07155	0.7915	2.815	0.2881	99.9431	98.8618	94.9635	LRRK2
Carbapenem	DB18912	0.7895	2.7894	0.3668	99.9349	98.6992	94.5795	LRRK2
Monoisopropylphosphorylserine	DB01805	0.7883	2.7748	0.2895	99.9268	98.5366	94.3604	LRRK2
TNP-2092	DB16312	0.8257	3.5258	0.3485	100.0	100.0	100.0	APOE
Tropatepine	DB13252	0.8048	3.267	0.3413	99.9919	99.8374	96.4046	APOE
Penfluridol	DB13791	0.7925	3.1144	0.3497	99.9837	99.6748	94.2841	APOE
2-PHENYLAMINO-4-METHYL -5-ACETYL THIAZOLE	DB08359	0.7856	3.0288	0.2741	99.9756	99.5122	93.0947	APOE
GW-590735	DB07215	0.7847	3.0171	0.3103	99.9675	99.3496	92.9328	APOE
Spiradoline	DB12704	0.7784	2.9386	0.3268	99.9593	99.187	91.8418	APOE
Ginsenoside Rg3	DB19257	0.7745	2.891	0.339	99.9512	99.0244	91.1806	APOE
2-[(2’,3’,4’-TRIFLUOROBIPHENYL -2-YL)OXY]ETHANOL	DB08611	0.7739	2.8836	0.3161	99.9431	98.8618	91.0774	APOE
6-(4-chloro-2-fluoro-3-phenoxybenzyl) pyridazin-3(2H)-one	DB08379	0.7732	2.8746	0.2895	99.9349	98.6992	90.9535	APOE
Palovarotene	DB05467	0.7674	2.8021	0.3271	99.9268	98.5366	89.9458	APOE
Chloroxylenol	DB11121	0.7924	3.3041	0.3651	100.0	100.0	100.0	FYN
(6-METHYL-3,4-DIHYDRO-2H -CHROMEN-2-YL)METHYLPHOSPHINATE	DB07487	0.7839	3.2009	0.35	99.9919	99.8374	98.5094	FYN
Soblidotin	DB12677	0.7825	3.1837	0.3229	99.9837	99.6748	98.2605	FYN
IOWH-032	DB12959	0.7802	3.1558	0.3662	99.9756	99.5122	97.8585	FYN
Felodipine	DB01023	0.7783	3.1326	0.2964	99.9675	99.3496	97.5222	FYN
Transcrocetinate	DB05974	0.775	3.0924	0.3199	99.9593	99.187	96.9417	FYN
Zolazepam	DB11555	0.7705	3.0375	0.3856	99.9512	99.0244	96.1488	FYN
(2S,3S)-3-AMINO-4-[(3S)-3-FLUOROPYRROLIDIN -1-YL]-N,N-DIMETHYL-4-OXO -2-(TRANS-4-[1,2,4]TRIAZOLO[1,5-A] PYRIDIN-5-YLCYCLOHEXYL)BUTANAMIDE	DB07135	0.769	3.0194	0.366	99.9431	98.8618	95.8886	FYN
Zuretinol acetate	DB12112	0.7688	3.0168	0.3115	99.9349	98.6992	95.851	FYN
5-(4-CHLORO-5-PHENYL-3-THIENYL) -1,2,5-THIADIAZOLIDIN-3-ONE 1,1-DIOXIDE	DB07134	0.7655	2.976	0.3377	99.9268	98.5366	95.2617	FYN
Sodium stibogluconate	DB05630	0.7811	3.1995	0.3156	100.0	100.0	100.0	GPNMB
2-[5-Methanesulfonylamino-2-(4-Aminophenyl) -6-Oxo-1,6-Dihydro-1-Pyrimidinyl] -N-(3,3,3-Trifluoro-1-Isopropyl -2-Oxopropyl)Acetamide	DB03202	0.7783	3.1639	0.2998	99.9919	99.8374	99.4792	GPNMB
Ilginatinib	DB12784	0.7731	3.0989	0.3404	99.9837	99.6748	98.5272	GPNMB
2-{[4-(TRIFLUOROMETHOXY)BENZOYL] AMINO}ETHYL DIHYDROGEN PHOSPHATE	DB07745	0.7716	3.0801	0.1946	99.9756	99.5122	98.252	GPNMB
Brotizolam	DB09017	0.7662	3.0133	0.3337	99.9675	99.3496	97.2749	GPNMB
Gunagratinib	DB18191	0.7659	3.0097	0.3445	99.9593	99.187	97.2221	GPNMB
D-tartaric acid	DB01694	0.7659	3.0092	0.2981	99.9512	99.0244	97.215	GPNMB
Citraconic acid	DB04734	0.7653	3.0017	0.3137	99.9431	98.8618	97.1049	GPNMB
VB-309	DB18539	0.765	2.9978	0.3683	99.9349	98.6992	97.0487	GPNMB
VP-14637	DB12195	0.7563	2.8891	0.3066	99.9268	98.5366	95.4582	GPNMB
Hydrolyzed Cephalothin	DB02247	0.8197	3.3124	0.3474	100.0	100.0	100.0	BST1
Oxytetracycline	DB00595	0.8004	3.0762	0.379	99.9919	99.8374	96.7303	BST1
Dyclonine	DB00645	0.7999	3.0701	0.3148	99.9837	99.6748	96.6471	BST1
N-(5-chloro-1,3-benzodioxol-4-yl) -6-methoxy-7-(3-piperidin-1-ylpropoxy) quinazolin-4-amine	DB07249	0.7991	3.0604	0.3241	99.9756	99.5122	96.5123	BST1
(METHYLPYRIDAZINE PIPERIDINE PROPYLOXYPHENYL)ETHYLACETATE	DB08013	0.7983	3.0505	0.3066	99.9675	99.3496	96.3748	BST1
Ghavamiol	DB02492	0.7973	3.0384	0.2712	99.9593	99.187	96.2078	BST1
Cryptoxanthin	DB15914	0.7925	2.979	0.2972	99.9512	99.0244	95.3855	BST1
Emetonium iodide	DB13769	0.7916	2.9692	0.2971	99.9431	98.8618	95.2496	BST1
Isocyanomethane	DB04337	0.7875	2.9187	0.3061	99.9349	98.6992	94.5519	BST1
5-(PARA-NITROPHENYL PHOSPHONATE) -PENTANOIC ACID	DB08296	0.7864	2.9055	0.3235	99.9268	98.5366	94.3682	BST1

**Table A4:** Top 10 drugs predicted by Multi-Modal GNN for five genes with highest FCI scores in Cluster 4.

Drug Name	DrugBank ID	Raw Score	Z-Score	Uncertainty	Percentile	Elite Percentile	Probability	Gene
Latamoxef	DB04570	0.8272	3.6216	0.3132	100.0	100.0	100.0	NUCKS1
N-[4-(2-CHLOROPHENYL)-1,3-DIOXO -1,2,3,6-TETRAHYDROPYRROLO [3,4-C]CARBAZOL-9-YL]FORMAMIDE	DB07226	0.8099	3.4112	0.2473	99.9919	99.8374	97.0681	NUCKS1
Acetic Acid Salicyloyl-Amino-Ester	DB03667	0.7999	3.2887	0.3413	99.9837	99.6748	95.3599	NUCKS1
DCFBC F-18	DB14772	0.7974	3.2579	0.3708	99.9756	99.5122	94.9308	NUCKS1
Etoposide toniribate	DB17255	0.7858	3.1171	0.2376	99.9675	99.3496	92.9677	NUCKS1
Batefenterol	DB12526	0.7837	3.0912	0.4028	99.9593	99.187	92.6068	NUCKS1
Enalaprilat	DB09477	0.7827	3.0786	0.3313	99.9512	99.0244	92.4319	NUCKS1
Acetylcysteine zinc	DB14479	0.779	3.034	0.2801	99.9431	98.8618	91.8091	NUCKS1
7-thionicotinamide-adenine -dinucleotide phosphate	DB01763	0.776	2.9968	0.3188	99.9349	98.6992	91.2906	NUCKS1
4-(2,4-Dimethyl-1,3-thiazol-5-yl) -N-[4-(trifluoromethyl)phenyl] -2-pyrimidinamine	DB02915	0.774	2.9726	0.2886	99.9268	98.5366	90.954	NUCKS1
Ruzinurad	DB19209	0.8245	3.5258	0.28	100.0	100.0	100.0	DYRK1A
LY-2300559	DB13016	0.821	3.4832	0.3023	99.9919	99.8374	99.3795	DYRK1A
Nerandomilast	DB18237	0.8104	3.3521	0.2565	99.9837	99.6748	97.4689	DYRK1A
Dodecyltrimethylammonium	DB02779	0.8083	3.3269	0.2834	99.9756	99.5122	97.102	DYRK1A
3-(6-HYDROXY-NAPHTHALEN -2-YL)-BENZO[D]ISOOXAZOL-6-OL	DB07236	0.7954	3.1681	0.3167	99.9675	99.3496	94.7897	DYRK1A
2’-Deoxyuridine	DB02256	0.7953	3.1675	0.3452	99.9593	99.187	94.7809	DYRK1A
Alpha-D-Galactose-1-Phosphate	DB02317	0.7947	3.1601	0.2815	99.9512	99.0244	94.6729	DYRK1A
Zk-806450	DB02112	0.7854	3.0455	0.3473	99.9431	98.8618	93.0045	DYRK1A
Mibolerone	DB11429	0.7833	3.0204	0.3261	99.9349	98.6992	92.6387	DYRK1A
(5R)-N,N-DIETHYL-5-METHYL -2-[(THIOPHEN-2-YLCARBONYL)AMINO] -4,5,6,7-TETRAHYDRO-1-BENZOTHIOPHENE -3-CARBOXAMIDE	DB08033	0.779	2.9674	0.2347	99.9268	98.5366	91.8665	DYRK1A
AZD-9977	DB15418	0.8003	3.3393	0.3033	100.0	100.0	100.0	PIK3CA
Rupintrivir	DB05102	0.7916	3.2311	0.3329	99.9919	99.8374	98.4824	PIK3CA
Algestone	DB18000	0.7852	3.1511	0.2734	99.9837	99.6748	97.3593	PIK3CA
(5S)-2-(Cyclooctylamino)-5-methyl -5-propyl-1,3-thiazol-4(5H)-one	DB07866	0.7799	3.0857	0.2671	99.9756	99.5122	96.4422	PIK3CA
N-({(3R,4R)-4-[(benzyloxy)methyl]pyrrolidin -3-yl}methyl)-N-(2-methylpropyl) benzenesulfonamide	DB07505	0.7797	3.0838	0.2617	99.9675	99.3496	96.4161	PIK3CA
4-[4-(4-Methyl-2-Methylamino-Thiazol -5-Yl)-Pyrimidin-2-Ylamino]-Phenol	DB04407	0.7788	3.0719	0.3167	99.9593	99.187	96.2493	PIK3CA
N-acetylsulfanilyl chloride	DB12337	0.7786	3.0698	0.2934	99.9512	99.0244	96.2189	PIK3CA
Florantyrone	DB08975	0.7718	2.9847	0.3435	99.9431	98.8618	95.0261	PIK3CA
Nateglinide	DB00731	0.7689	2.9488	0.3018	99.9349	98.6992	94.523	PIK3CA
Thioctic acid tromethamine	DB06253	0.7683	2.9424	0.3525	99.9268	98.5366	94.4332	PIK3CA
Ferrous ascorbate	DB14490	0.814	3.3529	0.2848	100.0	100.0	100.0	KANSL1
Ilepatril	DB06604	0.809	3.2914	0.2804	99.9919	99.8374	99.0953	KANSL1
N-Pyridoxyl-2-Methylalanine-5-Phosphate	DB04241	0.7919	3.0815	0.3613	99.9837	99.6748	96.0113	KANSL1
Didesmethylrocaglamide	DB15496	0.791	3.0701	0.3166	99.9756	99.5122	95.8439	KANSL1
4-Nitrophenyl Phosphate	DB04214	0.7902	3.0599	0.2966	99.9675	99.3496	95.6938	KANSL1
Apstatin	DB04092	0.782	2.96	0.3656	99.9593	99.187	94.2249	KANSL1
Odevixibat	DB16261	0.7805	2.9408	0.3857	99.9512	99.0244	93.9427	KANSL1
CRS-3123	DB12262	0.7804	2.9395	0.2526	99.9431	98.8618	93.9245	KANSL1
2-KETO-6-PHOSPHATE-D -GLUCONIC ACID, ALPHA -FURANOSE FORM	DB04663	0.7793	2.9267	0.2983	99.9349	98.6992	93.7364	KANSL1
Coproporphyrin I containing CO(III)	DB04423	0.778	2.9106	0.2759	99.9268	98.5366	93.4998	KANSL1
1-Hydroxyamine-2-Isobutylmalonic Acid	DB02326	0.8038	3.3643	0.2747	100.0	100.0	100.0	SETD1A
Vinflunine	DB11641	0.7996	3.3134	0.2182	99.9919	99.8374	99.2457	SETD1A
Sobetirome	DB07425	0.7993	3.3099	0.3475	99.9837	99.6748	99.1936	SETD1A
Ellagic acid	DB08846	0.7843	3.1277	0.2739	99.9756	99.5122	96.4966	SETD1A
Paeonol	DB16830	0.7745	3.0087	0.2397	99.9675	99.3496	94.7347	SETD1A
Cladribine	DB00242	0.7733	2.994	0.3192	99.9593	99.187	94.5164	SETD1A
[(2R,3S,4R,5R)-5-(4-acetonyl-3-carbamoyl -pyridin-1-ium-1-yl)-3,4-dihydroxy -tetrahydrofuran-2-yl]methyl [[(2R,3S,4R,5R)-5-(6-aminopurin-9-yl) -3,4-dihydroxy-tetrahydrofuran-2-yl] methoxy-hydroxy-phosphoryl] phosphate	DB02732	0.7688	2.9399	0.3621	99.9512	99.0244	93.7162	SETD1A
Canrenoic acid	DB09015	0.766	2.9063	0.3699	99.9431	98.8618	93.219	SETD1A
ACV tripeptide	DB02025	0.7629	2.869	0.3143	99.9349	98.6992	92.6663	SETD1A
alpha-Amyl cinnamaldehyde	DB14175	0.7624	2.8624	0.3083	99.9268	98.5366	92.569	SETD1A

**Table A5:** Top 10 drugs predicted by Multi-Modal GNN for five genes with highest FCI scores in Cluster 5.

Drug Name	DrugBank ID	Raw Score	z-Sscore	Uncertainty	Percentile	Elite Percentile	Probability	Gene
MMI-175	DB02378	0.8096	3.4908	0.2966	100.0	100.0	100.0	MCCC1
Gedatolisib	DB11896	0.7983	3.352	0.3406	99.9919	99.8374	98.0308	MCCC1
(1S)-2-[(2S,5R)-2-(AMINOMETHYL)-5 -ETHYNYLPYRROLIDIN-1-YL]-1 -CYCLOPENTYL-2-OXOETHANAMINE	DB07356	0.789	3.2374	0.3992	99.9837	99.6748	96.4038	MCCC1
N-(3-chlorophenyl)-N-methyl-2-oxo-3 -[(3,4,5-trimethyl-1H-pyrrol-2-yl)methyl] -2H-indole-5-sulfonamide	DB07369	0.7753	3.0683	0.4258	99.9756	99.5122	94.0031	MCCC1
Testosterone decanoate	DB16001	0.7739	3.0506	0.289	99.9675	99.3496	93.7512	MCCC1
N-Alpha-(2-Naphthylsulfonyl)-N(3-Amidino -L-Phenylalaninyl)-4-Acetyl-Piperazine	DB04125	0.7727	3.036	0.3411	99.9593	99.187	93.5443	MCCC1
Nemonoxacin	DB06600	0.766	2.953	0.3403	99.9512	99.0244	92.3665	MCCC1
Fludeoxyglucose	DB15107	0.7655	2.9465	0.2928	99.9431	98.8618	92.274	MCCC1
GSK-2881078	DB16888	0.7615	2.8972	0.2934	99.9349	98.6992	91.5738	MCCC1
Valspodar	DB11869	0.7581	2.8552	0.3053	99.9268	98.5366	90.9779	MCCC1
Zinc cation	DB14532	0.7966	3.285	0.274	100.0	100.0	100.0	WWOX
Cyproterone acetate	DB04839	0.7818	3.1064	0.3301	99.9919	99.8374	97.3973	WWOX
Norethandrolone	DB12787	0.7746	3.0186	0.2726	99.9837	99.6748	96.1187	WWOX
Lutein	DB00137	0.7739	3.0108	0.3759	99.9756	99.5122	96.0039	WWOX
Hydrocortisone acetate	DB14539	0.7716	2.9825	0.2526	99.9675	99.3496	95.5923	WWOX
Cytidine-5’-Monophosphate	DB03403	0.7698	2.9607	0.2689	99.9593	99.187	95.2748	WWOX
Brivudine	DB03312	0.7687	2.9482	0.2961	99.9512	99.0244	95.092	WWOX
THIOPHENE-2,5-DISULFONIC ACID 2 -AMIDE-5-(4-METHYL-BENZYLAMIDE)	DB07363	0.7674	2.9324	0.2946	99.9431	98.8618	94.8617	WWOX
2-Methyl-3-(2-Aminothiazolo)Propanal	DB03024	0.7664	2.9202	0.2721	99.9349	98.6992	94.6842	WWOX
Talinolol	DB11770	0.7652	2.9057	0.325	99.9268	98.5366	94.4735	WWOX
Acteoside	DB12996	0.7781	3.2404	0.3076	100.0	100.0	100.0	CCT3
Hypophosphite	DB04053	0.7778	3.2365	0.3599	99.9919	99.8374	99.9413	CCT3
(2S)-2-[(2,1,3-BENZOTHIADIAZOL-4 -YLSULFONYL)AMINO]-2-PHENYL-N -PYRIDIN-4-YLACETAMIDE	DB07568	0.776	3.2149	0.2205	99.9837	99.6748	99.6192	CCT3
Osalmid	DB16273	0.7715	3.1599	0.3294	99.9756	99.5122	98.7977	CCT3
Open Form of 2’-Deoxy-Ribofuranose -5’-Phosphate	DB04087	0.77	3.1406	0.3502	99.9675	99.3496	98.5106	CCT3
UK-390957	DB11719	0.7684	3.1212	0.3623	99.9593	99.187	98.2208	CCT3
SRA-737	DB16876	0.7674	3.1096	0.3314	99.9512	99.0244	98.047	CCT3
Iodo-Willardiine	DB02818	0.7649	3.079	0.3202	99.9431	98.8618	97.5903	CCT3
Protokylol	DB06814	0.7633	3.0585	0.3364	99.9349	98.6992	97.284	CCT3
XL-888	DB12981	0.7605	3.0253	0.3113	99.9268	98.5366	96.7882	CCT3
Candoxatrilat	DB11623	0.8117	3.4084	0.3816	100.0	100.0	100.0	CRLS1
Tivantinib	DB12200	0.8109	3.3979	0.3615	99.9919	99.8374	99.8439	CRLS1
(R)-tacrine(10)-hupyridone	DB04614	0.8096	3.3829	0.2907	99.9837	99.6748	99.6194	CRLS1
9(S)-HODE	DB07302	0.7971	3.228	0.2996	99.9756	99.5122	97.3065	CRLS1
GSK-2982772	DB16875	0.7934	3.1826	0.3392	99.9675	99.3496	96.6298	CRLS1
RU82197	DB03268	0.7882	3.118	0.2283	99.9593	99.187	95.6651	CRLS1
Sulthiame	DB08329	0.7774	2.9851	0.3236	99.9512	99.0244	93.6806	CRLS1
Naluzotan	DB05562	0.7746	2.951	0.3131	99.9431	98.8618	93.1709	CRLS1
EP-217609	DB18393	0.7741	2.944	0.2171	99.9349	98.6992	93.0661	CRLS1
Brefeldin A	DB07348	0.7731	2.9318	0.3294	99.9268	98.5366	92.885	CRLS1
25-desacetylrifapentine	DB15213	0.7947	3.31	0.3141	100.0	100.0	100.0	PMVK
Diethylcarbamazine	DB00711	0.7928	3.2864	0.3038	99.9919	99.8374	99.6637	PMVK
Dexepicatechin	DB19253	0.7925	3.2826	0.2872	99.9837	99.6748	99.6098	PMVK
5-ALPHA-PREGNANE-3-BETA -OL-HEMISUCCINATE	DB08510	0.7908	3.262	0.3144	99.9756	99.5122	99.3156	PMVK
L-Histidine Beta Naphthylamide	DB01938	0.7906	3.2594	0.3844	99.9675	99.3496	99.2781	PMVK
(5E)-14-CHLORO-15,17-DIHYDROXY -4,7,8,9,10,11-HEXAHYDRO-2 -BENZOXACYCLOPENTADECINE -1,12(3H,13H)-DIONE	DB08153	0.7792	3.12	0.318	99.9593	99.187	97.2896	PMVK
Indeglitazar	DB07724	0.7773	3.0965	0.271	99.9512	99.0244	96.9553	PMVK
Deoxycytidylyl-3’,5’-guanosine	DB03326	0.7708	3.0171	0.3722	99.9431	98.8618	95.8224	PMVK
Formaldehyde	DB03843	0.7693	2.9986	0.1982	99.9349	98.6992	95.5585	PMVK
Drostanolone	DB00858	0.7673	2.974	0.3675	99.9268	98.5366	95.208	PMVK
